# A novel inhibitor of the mitochondrial respiratory complex I with uncoupling properties exerts potent antitumor activity

**DOI:** 10.1038/s41419-024-06668-9

**Published:** 2024-05-02

**Authors:** Alaa Al Assi, Solène Posty, Frédéric Lamarche, Amel Chebel, Jérôme Guitton, Cécile Cottet-Rousselle, Renaud Prudent, Laurence Lafanechère, Stéphane Giraud, Patrick Dallemagne, Peggy Suzanne, Aurélie Verney, Laurent Genestier, Marie Castets, Eric Fontaine, Marc Billaud, Martine Cordier-Bussat

**Affiliations:** 1 Université Grenoble Alpes, Inserm U1055, Laboratoire de Bioénergétique Fondamentale et Appliquée (LBFA), Grenoble, France; 2 Cell death and Childhood Cancers Laboratory, Centre de Recherche en Cancérologie de Lyon (CRCL), INSERM U1052- CNRS UMR5286, Université Claude Bernard de Lyon1, Centre Léon Bérard, LabEx DEVweCAN, Institut Convergence Plascan, Lyon, France; 3 Centre International de Recherche en Infectiologie (Team LIB), Equipe labellisée La Ligue 2017 and 2023. Université Lyon, INSERM, U1111, Université Claude Bernard Lyon 1, Centre National de la Recherche Scientifique, UMR5308, ENS de Lyon, Lyon, France; 4 Laboratoire de biochimie et pharmacologie-toxicologie, Centre Hospitalier Lyon-Sud, Hospices Civils de Lyon, F-69495, Pierre Bénite, France. Laboratoire de Toxicologie, Faculté de pharmacie ISPBL, Université Lyon 1, 69373 Lyon, France; 5 Université Grenoble Alpes, Inserm U1209, CNRS UMR5309, Institute for Advanced Biosciences, Grenoble, France; 6 Center for Drug Discovery and Development, Synergie Lyon Cancer Foundation, Lyon, Cancer Research Center, Centre Léon Bérard, Lyon, France; 7 Normandie Univ., UNICAEN, CERMN, 14000 Caen, France

**Keywords:** Biologics, Cancer metabolism

## Abstract

Cancer cells are highly dependent on bioenergetic processes to support their growth and survival. Disruption of metabolic pathways, particularly by targeting the mitochondrial electron transport chain complexes (ETC-I to V) has become an attractive therapeutic strategy. As a result, the search for clinically effective new respiratory chain inhibitors with minimized adverse effects is a major goal. Here, we characterize a new OXPHOS inhibitor compound called MS-L6, which behaves as an inhibitor of ETC-I, combining inhibition of NADH oxidation and uncoupling effect. MS-L6 is effective on both intact and sub-mitochondrial particles, indicating that its efficacy does not depend on its accumulation within the mitochondria. MS-L6 reduces ATP synthesis and induces a metabolic shift with increased glucose consumption and lactate production in cancer cell lines. MS-L6 either dose-dependently inhibits cell proliferation or induces cell death in a variety of cancer cell lines, including B-cell and T-cell lymphomas as well as pediatric sarcoma. Ectopic expression of *Saccharomyces cerevisiae* NADH dehydrogenase (NDI-1) partially restores the viability of B-lymphoma cells treated with MS-L6, demonstrating that the inhibition of NADH oxidation is functionally linked to its cytotoxic effect. Furthermore, MS-L6 administration induces robust inhibition of lymphoma tumor growth in two murine xenograft models without toxicity. Thus, our data present MS-L6 as an inhibitor of OXPHOS, with a dual mechanism of action on the respiratory chain and with potent antitumor properties in preclinical models, positioning it as the pioneering member of a promising drug class to be evaluated for cancer therapy.

**Figure:**
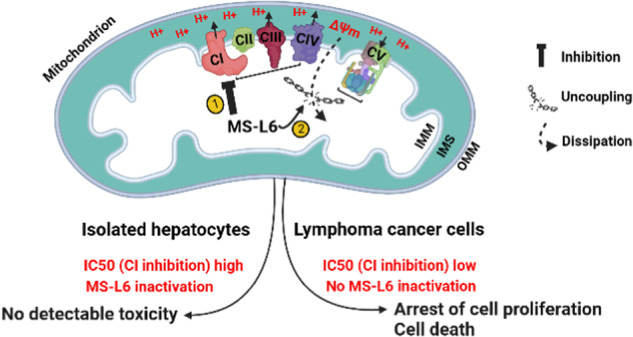
MS-L6 exerts dual mitochondrial effects: ETC-I inhibition and uncoupling of OXPHOS. In cancer cells, MS-L6 inhibited ETC-I at least 5 times more than in isolated rat hepatocytes. These mitochondrial effects lead to energy collapse in cancer cells, resulting in proliferation arrest and cell death. In contrast, hepatocytes which completely and rapidly inactivated this molecule, restored their energy status and survived exposure to MS-L6 without apparent toxicity.

MS-L6 exerts dual mitochondrial effects: ETC-I inhibition and uncoupling of OXPHOS. In cancer cells, MS-L6 inhibited ETC-I at least 5 times more than in isolated rat hepatocytes. These mitochondrial effects lead to energy collapse in cancer cells, resulting in proliferation arrest and cell death. In contrast, hepatocytes which completely and rapidly inactivated this molecule, restored their energy status and survived exposure to MS-L6 without apparent toxicity.

## Introduction

The transformation, development and spread of malignant cells are driven by and dependent on metabolic adaptation [1–3]. ATP is notably produced by mitochondria through oxidative phosphorylation (OXPHOS) which is often upregulated during the progression of a variety of adult and pediatric cancers [4], including hematological malignancies, such as lymphoma [5] and leukemia [6–8], but also solid tumors such as sarcoma [9, 10]. ATP production by mitochondria involves three steps [11]: i) the catabolism of carbohydrates, lipids and amino acids into NADH + H^+^ and FADH_2_, CO_2_ and NH_3_, ii) the oxidation of NADH + H^+^ and FADH_2_ by the MRC (electron transport chain complexes ETC-I to IV) located in the inner mitochondrial membrane (IMM), resulting in an electron flux (the consumed oxygen acting as a terminal electron acceptor) and the expulsion of protons contributing to the generation of the proton gradient and iii) the consumption of the proton gradient by the ATPase leading to the synthesis of ATP from ADP and phosphate (Fig. S1) [12]. Measurement of the oxygen consumption rate (OCR) indirectly reflects OXPHOS activity [13]. Although tumorigenesis depends on functional OXPHOS, OXPHOS-derived ATP is not always required [14] and is produced at a slower rate in solid tumors than in corresponding healthy tissues in preclinical mouse models [15]. However, targeting OXPHOS pathways is a pursued strategy for the development of antitumoral drugs [4, 16]. Several modulators of OXPHOS have been described, acting as inhibitors of either electron or proton transport. These include potent inhibitors targeting ETC-1 and uncouplers. Several ETC-1 inhibitors (rotenone, metformin, IACS-010759, and IM156) inhibit NADH oxidation and electron transfer in the MRC [17], while uncouplers (FCCP and BAM-15) mediate proton transport into the matrix and dissipate the proton gradient [18]. Others, including MitoTAM, combine these properties [19]. They all reduce mitochondrial ATP production.

The clinical success of the anti-diabetic drug metformin first suggested the relevance of an anti-metabolic pharmacological approach in the treatment of cancer [20]. Indeed, epidemiological studies have shown that its widespread use reduces cancer incidence in diabetic patients [21, 22], although the magnitude of this effect has been debated [23]. This observation has led to a strong interest in ETC-I inhibitors for cancer therapy. Clinical trials are underway to evaluate the efficacy of several OXPHOS inhibitors in the prevention and treatment of various types of cancer [24]. Several of these inhibitors including IACS-010759 [25] have recently been shown to have compromising side effects, while other molecules such as IM156 and MitoTAM have successfully completed stage I clinical trials [26, 27]. In this context, the identification of new inhibitors remains essential to effectively mitigate the adverse side effects of new drug candidates [28, 29]. Here, we characterized a novel molecule, MS-L6, targeting OXPHOS with a dual mechanism of action. MS-L6 exhibits mild cytotoxic effects on several cancer cell lines and remarkably inhibits tumor growth in preclinical mouse cancer models without toxicity. These results provide evidence for a new class of OXPHOS modulators that should be further explored for drug development.

## Results

### MS-L6 inhibits respiration through ETC-I

**Fig. 1:**
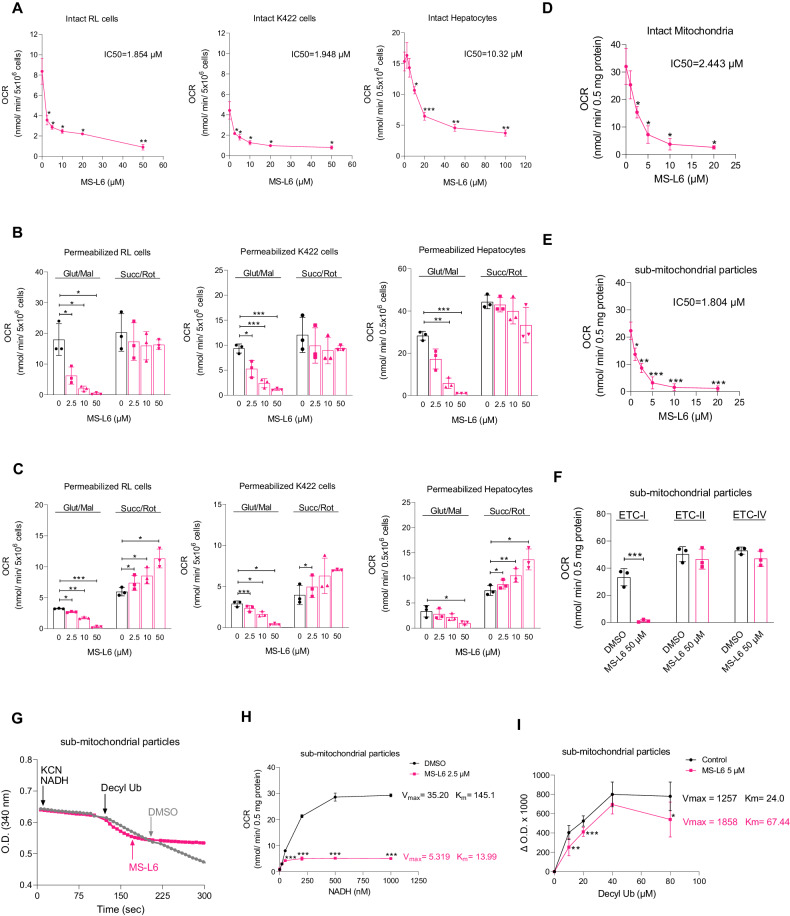
MS-L6 inhibits respiration of hepatocytes and cancer cells via ETC-I. **A** Shows the OCR of intact hepatocytes, RL and K422 cells as function of MS-L6 concentration. The corresponding IC_50_ values of MS-L6 were calculated by fitting of dose-response curves in GraphPad Prism and are indicated above each curve. Data are presented as mean ± SD, *n* = 3. **B**, **C** Show the OCR of digitonin-permeabilized hepatocytes, RL and K422 cells treated with the indicated concentrations of MS-L6, driven by ETC-I (Glut/Mal :5 mM malate, 2.5 mM glutamate) or ETC-II (Succ/Rot:5 mM succinate, 1 µM rotenone) substrates in mitochondrial assay buffer, under ADP phosphorylation conditions (5 mM ADP) and resting state (1 µM oligomycin), respectively. Data are shown as means ± SD, *n* = 3. Panel (**D**) reports OCR of 0.5 mg/mL of intact rat liver mitochondria driven by ETC-I substrate (Glut/Mal) as function of MS-L6 concentration under phosphorylation conditions. Data are presented as mean ± SD, *n* = 3. **E** Shows OCR of 0.5 mg/mL of rat liver sub-mitochondrial particles driven by ETC-I substrate (1 mM NADH, in the presence of 5 mM inorganic phosphate) as function of MS-L6 concentration. Data are expressed as mean ± SD, *n* = 3. **F** Shows OCR of 0.5 mg/mL rat liver sub-mitochondrial particles driven by substrates for ETC-I (1 mM NADH), ETC-II (2 mM succinate) and ETC-IV (250 µM/100 µM TMPD/ascorbate), respectively, in the presence of DMSO (vehicle control) or 50 µM MS-L6. Data are expressed as mean ± SD, *n* = 3. **G** Shows ETC-I activity as measured by following NADH absorbance by spectrophotometry at 340 nM of rat liver sub-mitochondrial particles in the presence of DMSO (vehicle control) or 50 µM MS-L6. Sub-mitochondrial particles were first incubated with 100 µM NADH in the presence of 1 mM KCN, and then 100 µM decylubiquinone was added. Where indicated, DMSO (black lane) or 50 µM MS-L6 (pink lane) was added. The absorbance curves represent one typical experiment; similar results were obtained in two other experiments. **H** Shows the OCR of 0.5 mg/mL sub-mitochondrial particles incubated with DMSO (vehicle control) or 2.5 µM MS-L6, after titration with increasing concentrations of the ETC-I substrate, NADH. Maximum velocity (Vmax) and Michaelis constant (Km) of each lane are indicated. Data are presented as mean ± SD, *n* = 3. **I** Reports ETC-I activity measured by following NADH absorbance spectrophotometrically at 340 nM of rat liver sub-mitochondrial particles in the presence of DMSO (vehicle control) or 5 µM MS-L6. Sub-mitochondrial particles were incubated with 1 mM NADH in the presence of 1 mM KCN upon titration with increasing concentrations of decylubiquinone. Because higher concentration of decylubiquinone tended to inhibit NADH oxidation, especially in the presence of MS-L6(Fig. S2A left), we constructed Michaelis-Menten saturation curves showing the NADH oxidation of mitochondrial fragments incubated with either DMSO or MS-L6, upon titration with increasing concentrations of decylubiquinone by removing the higher concentration. Maximum velocity (Vmax) and Michaelis constant (Km) of each trace are shown. Data are expressed as means ± SD, *n* = 3. Statistical significance was determined as described in materials and methods.

MS-L6 is a synthetic bis-thioureidic derivative with anti-amoebic properties [30, 31] and has emerged from a chemical library screen aimed at identifying compounds that could induce cell death and interfere with metabolic pathways. Its chemical structure is distantly related to diguanidine compounds (Fig. S1), such as the metformin ETC-I inhibitor [32]. Therefore, we investigated the effects of MS-L6 on the OCR of freshly isolated rat hepatocytes and two lymphoma cell lines, namely RL and K422, by titration with increasing concentrations. MS-L6 dose-dependently decreased the OCR of the 3 cell types (Fig. 1A), but the IC_50_ was 5-fold higher in hepatocytes. Given that complete OCR inhibition was always observed at 50 µM, we were prompted to further analyze the effects of MS-L6 on mitochondria at this concentration.

To determine on which complex of MRC MS-L6 acts on, we examined its effects on cells permeabilized with low concentrations of digitonin to remove cholesterol from plasma membranes and exposes mitochondria to extracellular metabolic substrates added to the experimental buffer. Under ADP-phosphorylating conditions (state 3/ATP synthase is active), MS-L6 reduced the OCR of permeabilized hepatocytes, RL and K422 cells in the presence of ETC-I substrate (glutamate/malate), but not in the presence of ETC-II substrate (succinate) and rotenone, highlighting its specificity for ETC-I (Fig. 1B).

Under non-phosphorylating conditions (state 4/resting OXPHOS) achieved by the addition of the ATP synthase inhibitor oligomycin, MS-L6 decreased residual glutamate/malate driven OCR (ETC-I-dependent) and increased succinate/rotenone driven OCR (ETC-I-independent) in concentration dependent manner in all cells tested (Fig. 1C). This suggests that MS-L6 also acts as an uncoupler of OXPHOS, typically by inducing a process that consumes oxygen without leading to ATP synthesis.

We next tested whether MS-L6-induced inhibition of ETC-I was dependent on the integrity of the mitochondrial membrane potential ΔΨm. Since sub-mitochondrial particles lack ΔΨm, we compared ETC-I-driven OCR of intact mitochondria with that of sub-mitochondrial particles, which were stimulated with glutamate/malate and NADH respectively. MS-L6 decreased the OCR of both intact mitochondria and sub-mitochondrial particles with similar IC_50_ values (Fig. 1D, E), indicating that MS-L6 inhibited ETC-I-driven OCR regardless of mitochondrial membrane integrity, ΔΨm maintenance or matrix accumulation.

To confirm that ETC-I was the target of MS-L6, we evaluated the effects of MS-L6 on the OCR of sub-mitochondrial particles specifically powered through ETC-I, -II, and -IV using NADH, succinate and TMPD/ascorbate substrates, respectively. Again, MS-L6 exclusively inhibited OCR through ETC-I (Fig. 1F). Finally, we measured NADH oxidation by ETC-I which was functionally isolated from the rest of the MRC by incubating sub-mitochondrial particles with NADH in the presence of KCN to block electron transfer by ECT-IV. When decylubiquinone was added at a constant concentration as the final acceptor of electrons from NADH oxidation, MS-L6 completely blocked NADH oxidation (Fig. 1G). We also constructed Michaelis-Menten saturation curves showing the OCR of sub-mitochondrial particles incubated with either DMSO or MS-L6, upon titration with increasing concentrations of NADH. The maximal OCR (Vmax) and NADH concentrations at half-maximal OCR (Km) decreased in the presence of MS-L6, indicating that MS-L6 inhibited ETC-I without competing with NADH (Fig. 1H). On the contrary, with a constant concentration of NADH and increasing concentrations of decylubiquinone, we observed a clear competition as if decylubiquinone could displace MS-L6 from its binding site (Figs. 1I, S2A).

### MS-L6 acts on ΔΨm

**Fig. 2:**
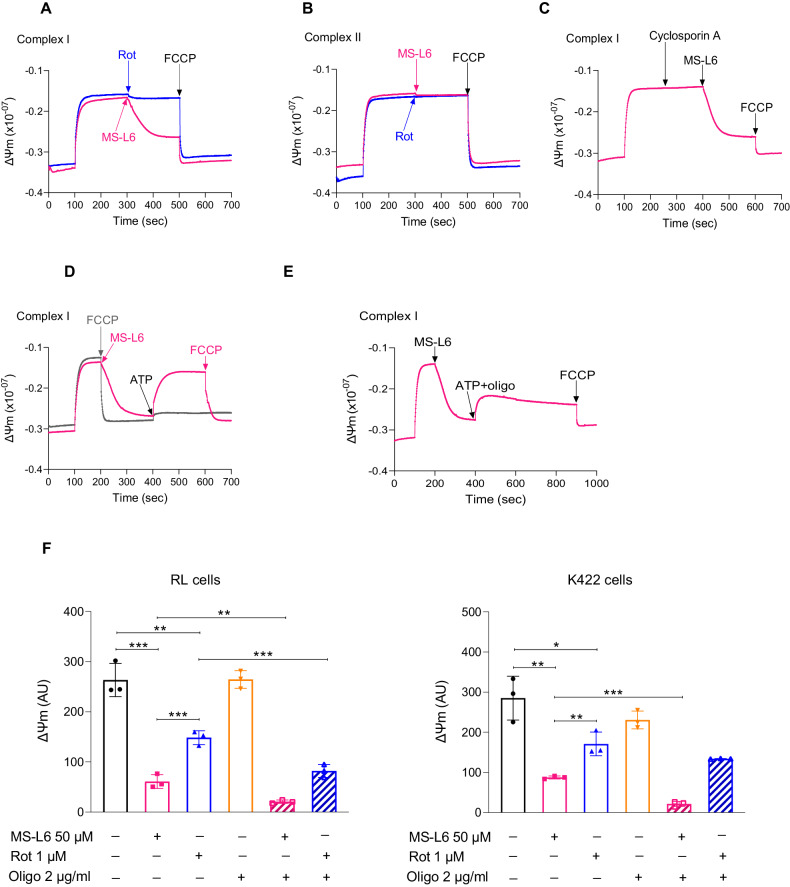
MS-L6 decreases ΔΨm in rat liver mitochondria and cancer cells. **A**–**C** Report ΔΨm of 0.5 mg/mL of intact rat liver mitochondria energized with ETC-I substrate (Glut/Mal), with ETC-II substrate (Succ), or with ETC-I substrate (Glut/Mal) after cyclosporin A (CsA) pretreatment, respectively, after treatment with either 1 µM rotenone (blue curve) or 50 µM MS-L6 (pink curve). Finally, 0.875 µM FCCP was added to fully depolarize ΔΨm. **D***,* **E** Show ΔΨm of 0.5 mg/ml of intact rat liver mitochondria energized with Glut/Mal, after treatment with 50 µM MS-L6 or 0.875 µM FCCP followed by addition of ATP, or with 50 µM MS-L6 followed by addition of ATP plus 2 µg/ml oligomycin, respectively. All curves illustrate one typical experiment and similar results were obtained in 2 other experiments. **F** Shows ΔΨm of intact RL and K422 cells, measured 1 h after treatment with DMSO (negative control), or different combinations of 50 µM MS-L6, 1 µM rotenone and 2 µg/mL oligomycin. Data are expressed as mean ± SD, *n* = 3. Statistical significance was determined as described in materials and methods.

Our finding that MS-L6 increased succinate/rotenone driven OCR in Fig. 1C led us to hypothesize that MS-L6 may be endowed with uncoupling properties. We therefore investigated its effect on ΔΨm. In intact freshly isolated mitochondria energized with ETC-I substrate, MS-L6 reduced ΔΨm (Fig. 2A). When incubated with rotenone, an ETC-I inhibitor lacking uncoupling properties at a dose that completely inhibits OCR, only a small decrease in ΔΨm was observed. Subsequent addition of the very low doses of the protonophore uncoupler FCCP completely depolarized ΔΨm (Fig. S2B–D). The residual ΔΨm after addition of rotenone was higher than that observed with MS-L6 (Fig. 2A) and persisted in the absence of BSA (Fig. S2B), indicating that MS-L6 alone induced a stronger depolarization than rotenone. MS-L6 caused a small yet noticeable decrease in ΔΨm (comparable to 100 nM FCCP (Fig. S2C) in the presence of ETC-II substrate, whereas rotenone had no effect (Fig. 2B). As expected, higher concentrations of FCCP completely abolished ΔΨm (Fig. S2C). Notably, pre-incubation with the permeability transition pore (PTP) cyclosporin A inhibitor did not attenuate MS-L6 effect in the presence of ETC-I substrate, suggesting that it was not mediated by PTP opening (Fig. 2C). Finally, we compared the effect of 50 µM MS-L6 to 100 nM FCCP on the oxygen consumed to synthetize the same amount of ATP in the presence of ECT-II substrate. As depicted in Fig S2C right, MS-L6 and FCCP increased the amount of oxygen required for ATP synthesis further confirming the uncoupling effect of MS-L6.

ATP synthase (ECT-V) uses the free energy of the electrochemical proton gradient generated by the MRC to synthesise ATP. ATP synthase is a reversible proton pump that can also hydrolyze ATP to restore ΔΨm. We questioned whether the uncoupling effect of MS-L6 could be compensated for by reversing the activity of ATP synthase. This “ATP hydrolyzing” activity of ATP synthase was switched on by the addition of ATP to intact mitochondria energized with ETC-I substrate, after MS-L6 (50 µM) or FCCP (875 nM) induced depolarization, Addition of ATP restored ΔΨm following MS-L6 treatment but not FCCP treatment (Fig. 2D). To further confirm that the ATP-induced compensation of ΔΨm was mediated via ATP synthase, we added ATP in combination with oligomycin, an irreversible inhibitor of ATP synthase. A small restoration of ΔΨm was observed, confirming that ATP hydrolysis by ATP synthase compensated for MS-L6-induced depolarization (Fig. 2E). We next measured the effects of MS-L6 on the ΔΨm in intact cancer cells. Compared to untreated cells, MS-L6 decreased ΔΨm by at least twice as much as rotenone, in both RL and K422 cells (Fig. 2F). Knowing that oligomycin alone had no effect on ΔΨm, the co-treatment of cancer cells with MS-L6 and oligomycin resulted in a greater decrease in ΔΨm than treatment with MS-L6 alone. Similar trends were observed with cotreatment of rotenone and oligomycin.

All these results confirm that MS-L6, in addition to inhibiting ETC-I, also has an uncoupling effect.

### MS-L6 modifies the energy and metabolic status in cancer cells

**Fig. 3:**
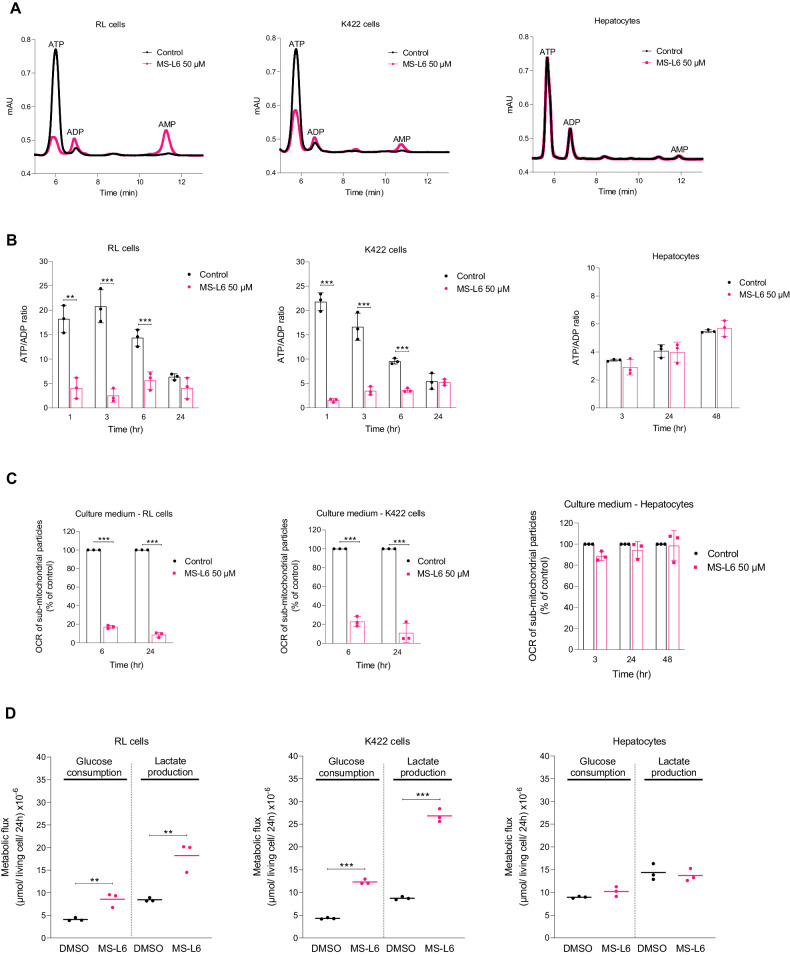
MS-L6 modifies the energy status in cancer cells. **A** Represents typical HPLC chromatograms showing the peaks of ATP, ADP and AMP of entire hepatocytes, RL and K422 cells cultured in the presence of DMSO (vehicle control) or 50 µM MS-L6 up to 3 h. **B** Represents calculated ATP/ADP ratios obtained after 3, 24, and 48 h of treatment. Data are presented as mean ± SD, *n* = 3. **C** Shows OCR of rat liver sub-mitochondrial particles driven by ETC-I substrate (1 mM NADH) in the presence of culture medium from hepatocytes, RL and K422 cells at different time points after treatment with 50 µM MS-L6 compared to culture medium from control cells (% of control). Data are presented as means ± SD, *n* = 3. **D** Reports glucose consumption and lactate production in hepatocytes, RL and K422 cells cultured in the presence of either DMSO (vehicle control) or 50 µM MS-L6 for 48 h. Metabolic fluxes are expressed as number of units (µmol) per unit of living cells per 24 h. The mean is shown, *n* = 3. Statistical significance was determined as described in materials and methods.

We investigated the effects of MS-L6 treatment on cellular energy status. The total adenine nucleotide content was measured by HPLC, resulting in chromatograms showing the respective ATP, ADP and AMP peaks (Fig. 3A) and the ATP/ADP ratios were calculated (Fig. 3B). As expected for an ETC-1 inhibitor, MS-L6 treatment dramatically reduced the ATP/ADP ratios in RL and K422 cells. Surprisingly, hepatocytes treated with 50 µM MS-L6 had ATP/ADP ratios like those of untreated cells, regardless of treatment duration. Since MS-L6 was administered at a dose that completely inhibited OCR in all cell types, we questioned its availability in the culture medium. We collected media at different time points and tested their effects on NADH-driven OCR of sub-mitochondrial particles. Media collected 6 and 24 h after treatment of either RL or K422 cells strongly inhibited NADH-driven OCR of sub-mitochondrial particles (Fig. 3C). In contrast, media collected as early as 3 h after treatment of hepatocytes had very little or no inhibitory effect on OCR (Fig. 3C). Preincubation of MS-L6 for 3 h at 37 °C in culture media without hepatocytes did not subsequently decrease MS-L6 activity (Fig. S3A), suggesting that hepatocytes metabolize MS-L6.

Inhibition of OXPHOS often induces a metabolic shift towards glycolysis, characterized by increased cellular glucose consumption and lactate production. These two parameters were effectively increased in RL and K422 cells after 24 h treatment with MS-L6 (Fig. 3D), but not in hepatocytes as expected. Accordingly, real-time metabolic analysis using the Seahorse technology confirmed this glycolytic shift, as manifested by an increase of the extracellular acidification rate (ECAR) and decrease of the OCR following MS-L6 treatment like IACS-010759 or rotenone treatment, although more progressively and to a lesser extent (Fig. S3B).

### Comparative in vitro analysis of MS-L6 toxicity

We evaluated MS-L6 toxicity by measuring its effect on the number of viable cells. We observed a similar decrease between treated and untreated hepatocytes (Fig. S4A–C). Since it is common that 50–70% of hepatocytes in primary cultures die progressively, we concluded that MS-L6 treatment did not affect the viability of hepatocytes. On the contrary, 50 µM MS-L6 completely blocked the proliferation of RL (the number of live cells remained unchanged over time) and killed K422 cells (the number of live cells decreased over time) (Fig. S4D, E). Correspondingly, flow cytometric analysis revealed mild and extensive death, highlighted by Annexin V/PI co-labeling of RL and K422 cells, respectively, after treatment (Fig. S5F).

**Fig. 4:**
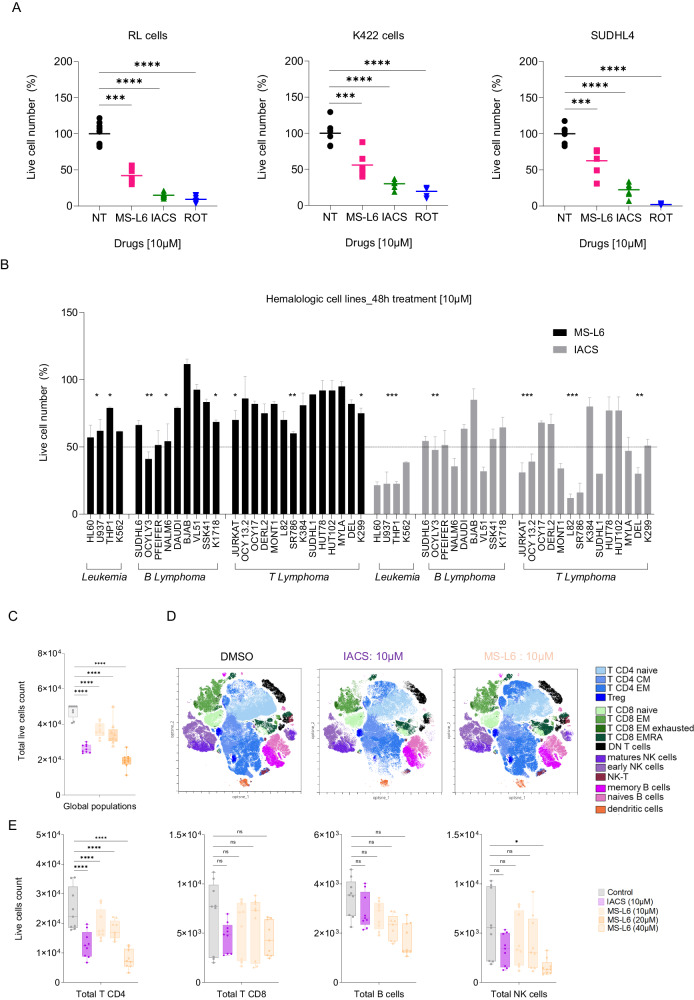
MS-L6 toxicity on tumour versus non tumour cells. **A** Shows the number of live cells; automated flow cytometry analysis allows total and live cell count per well. Analysis was performed 48 h after treatment of RL, K422 and SUDHL4 cells with DMSO diluent (NT), 10 µM MS-L6, 10 µM IACS-0105-759 or 1 µM rotenone. To standardize the analyses between cell lines and experiments, the mean of the number of live cells in 8 replicate wells treated with diluent (NT) was calculated and then the percentage of live cells post-treatment relative to this mean was calculated. Each point represents the percentage obtained for one replicate well. Line represents the mean of values. The graph is representative of 3 independent experiments. **B** Shows the percentage of viable cells detected 48 h after treatment with 10 µM MS-L6 or IACS-010759 in a panel of malignant haematological cell lines. The percentage of viable cells treated with MS-L6/IACS-010759 compared to cells treated with diluent is shown, calculated as in (**A**). Data are presented as mean ± SD of 3 independent experiments with at least 4 technical replicates. Significance has been indicated for the most representative cell lines only, to make the graph easier to read. **C**–**E** Represent analysis of MS-L6 effects on human PBMCs using flow cytometric immunophenotyping. **C** Shows the global number of live cells (CD45 + /annexin), i.e. including all populations, according to the treatment applied to the cells (diluent: DMSO, IACS-010759 10 µM and MS-L6 10-40 µM). **D** Shows the optimised t-SNE representation after integration of the 3 donor data sets with visualisation of all PBMC subpopulations. In this representation, each dot represents one cell, and each colour represents one subpopulation identified by labelling with the antibodies described in Table S1. Individual donor data sets are available on (Fig. S5). **E** Shows the live cell counts of the more visually affected populations in the panel (**D**), with each point on boxes and whiskers (Min to Max, all points) representing the value of a single technical replicate, for each of the 3 donor samples described in the figure S5. Statistical significance was determined as described in materials and methods.

We then compared the cytotoxic effects of MS-L6 at a lower dose with those of IACS-010759, on a panel of human cell lines. In drug candidate screening, the 10 µM concentration is conventionally considered the upper limit [33]. We chose to evaluate the effects of MS-L6 at 10 µM which is the concentration that reduced approximately 80% of the OCR in RL and K422 cells (Fig. 1). Live and dead cells were quantified by automated flow cytometry, initially in RL, K422 and SUDHL4 cells. The number of live cells decreased by 50% with MS-L6 in the three cell lines, although to a lesser extent than with IACS-010759 tested at the same dose or rotenone (Fig. 4A). Annexin V/PI labeling showed that MS-L6 induced moderate cell death exclusively in K422 cells, whereas IACS-010759 and rotenone killed all three cell lines (Fig. S4H). Comprehensive analysis of malignant haematological cell lines confirmed this observation: treatment with 10 µM MS-L6 resulted in a reduction in the number of viable cells, with varying degrees, ranging from moderate to comparable to the effect of IACS-010759 (Fig. 4B).

The toxicity of MS-L6 on non-tumoral lymphocytes was then evaluated. We compared the effects of increasing concentrations (10–40 µM) of MS-L6 with those of 10 µM IACS-010759 on the survival of fresh peripheral blood mononuclear cells (PBMCs) from 3 healthy donors using flow cytometry immunophenotyping (Figs. 4C–E, S5, Table S1). The global live cell count, represented by CD45 + /annexin- cells, 24 h post-treatment with 10–20 µM MS-L6 was higher than that observed with 10 µM IACS-100759 (Fig. 4C). Optimized t-SNE integration [34] of data sets from the 3 donors revealed that the 10 µM MS-L6 treatment had an overall smaller effect than the 10 µM IACS-010759 treatment on most of the CD45 positive subsets examined (Figs. 4D, S5A), especially on total CD4+ and CD8 + T lymphocytes, and NK cells (Figs. 4E, S5B). The number of B lymphocytes was not significantly decreased. The significantly reduced cell subsets were T CD4+ naive cells at the higher dose of MS-L6 treatment (40 µM). These results demonstrate that the toxicity of MS-L6 on PBMC-derived lymphocytes is less than or equivalent to that observed with IACS-010759 at similar concentrations.

Finally, we confirmed the toxicity of MS-L6 on several solid tumor cell lines, in particular the pediatric rhabdomyosarcoma cell lines RH30 and RD (Fig. S6A). Real-time imaging with fluorescent dyes confirmed that in these cell types (Fig. S6B) as well as in B lymphoma (Fig. S6C) MS-L6 is (i) mostly cytostatic at a moderate dose (10 µM) where fewer but viable cells are highlighted by green labeling and (ii) mostly cytotoxic at a high dose (50 µM) where most of the remaining cells are dead, highlighted by red labeling.

### Functional link between MS-L6 treatment and cytotoxicity

To determine whether MS-L6 inhibition of ETC-I is responsible for its cytotoxic properties, we performed complementation experiments with the *Saccharomyces cerevisiae* NDI-1 protein. NDI-1 oxidizes NADH like the mammalian multiunit ETC-I but without pumping protons into the intermembrane space [35, 36] and is resistant to most ETC-I described inhibitors.

**Fig. 5:**
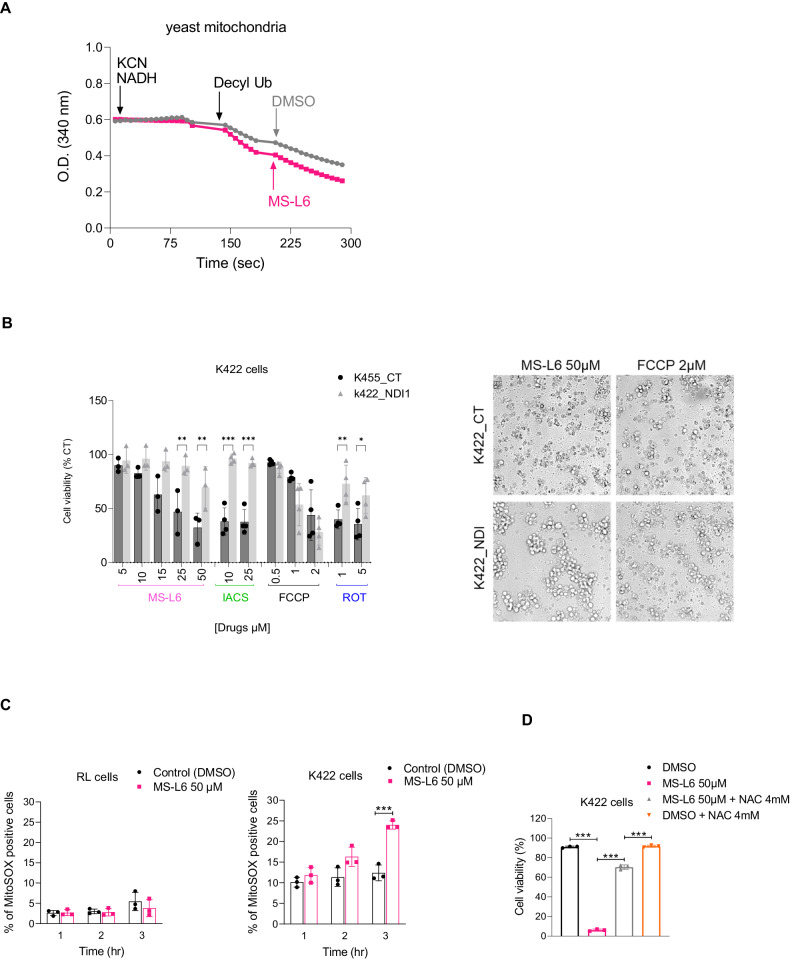
ECT-1 targeting by MS-L6 causes tumor cell toxicity. **A** Reports effect of MS-L6 on NADH oxidation activity (as shown in Fig. 1G) of yeast mitochondria. NADH absorbance was measured spectrophotometrically at 340 nM. NDI-1 was functionally isolated from the rest of the MRC by incubating yeast mitochondria with 100 µM NADH substrate, 1 mM KCN to block electron transfer by ECT-IV and 100 µM decylubiquinone as the final acceptor of electrons from ECT-I. Then, where indicated, DMSO (black trace) or 50 µM MS-L6 (pink trace) was added. These curves represent one typical experiment; similar results were obtained in two other experiments. **B** Reports the viability of K422 cells overexpressing (NDI1) or not (CT) the yeast NDI1 protein, after treatment with increasing doses of MS-L6, IACS-010759, FCCP or rotenone. Results are expressed as percentage of cells treated with vehicle (DMSO). Viable cells were detected using CellTiter-Fluor™ Cell Viability Assay. Each point represents the mean of 4 experimental replicates in one representative experiment, *n* = 3. Images of microscopic analysis of cells prior to biochemical assay are shown on the right. **C** Depicts the superoxide production of RL and K422 cells measured by flow cytometry using the MitoSOX probe. Data are presented as mean ± SD, *n* = 3. **D** Reports cell viability of K422 cells measured by trypan blue exclusion using the Lunaautomated cell counter (Logos Biosystem) exposed or not to 50 µM MS-L6 in the presence or absence of 4 mM *N*-acetylcysteine (NAC). Data are presented as mean ± SD, *n* = 3. Statistical significance was determined as described in materials and methods.

We verified that the yeast NADH-Q oxidoreductase NDI-1 was resistant to MS-L6 treatment by an indirect validation approach. As previously performed in Fig. 1G, we functionally isolated NDI-1 using yeast sub-mitochondrial particles and observed that the addition of MS-L6 did not block NADH oxidation (Fig. 5A), indicating that electron transfer by NDI-1 was not inhibited by MS-L6. We then evaluated the effect of MS-L6 treatment in K422 cells stably expressing the NDI-1 protein compared to their counterparts without NDI-1 expression (Fig. S7). As expected, NDI-1 expression restored the viability of K422 cells treated with IACS-010759 or rotenone used as positive controls for ETC-1 inhibitors but did not alter the viability of cells treated with FCCP uncoupler (Fig. 5B). A partial restoration of viability was observed in response to increasing doses of MS-L6, indicating that inhibition of ETC-I did contribute to K422 cell death.

We further found that MS-L6 did not induce ROS production in RL cells, whereas it did in KARPAS cells (Fig. 5C). Pretreatment of K422 cells with the antioxidant NAC prevented this effect (Fig. 5D) suggesting that cytotoxicity occurs in cells because of MS-L6-induced production of ROS.

### MS-L6 exhibits antitumor activity in preclinical mouse models

**Fig. 6:**
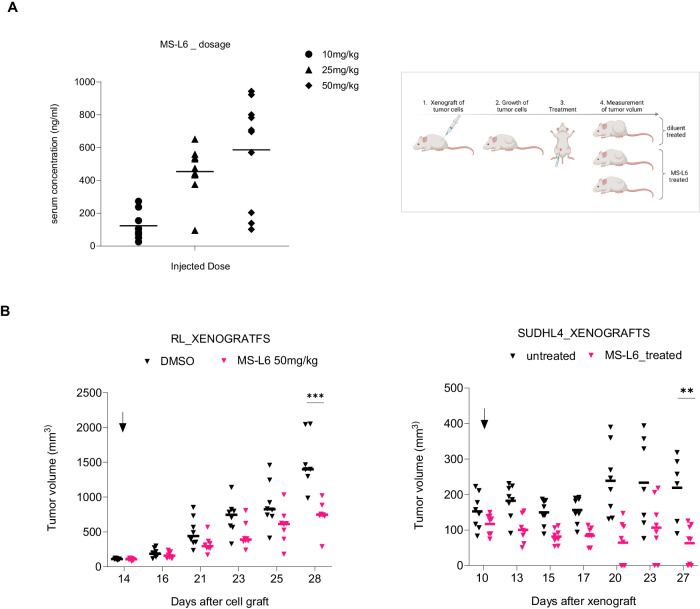
MS-L6 exerts antitumor activity in preclinical models. **A** Shows the dosage of MS-L6 in the sera of mice during preliminary pilot toxicity experiments using LC/MS analysis, 1 h after IP injection of increasing doses of the molecule (left), and the short procedure used for the preclinical evaluation of the effect MS-L6 in CDX models (right). The serum concentration reaches ~1 µM 1 h post-treatment with 50 mg/kg injected. After xenograft of human lymphoma cell lines in SCID mice, animals with nascent tumours were treated with diluent (DMSO) or 50 mg/kg MS-L6 by IP injection, 5/7 days per week, until the maximal ethically accepted volume was reached. **B** Reports the evolution of tumour volume of mice bearing subcutaneous xenografts of RL (left) or SUDHL4 (right) cells, following treatment with MS-L6 (50 mg/kg) or diluent (untreated). Median is shown. Each point represents one mouse. Statistical significance was determined as described in materials and methods.

We evaluated the antitumor activity of MS-L6 in preclinical SCID mouse models. Pilot experiments were conducted to evaluate the toxicity of MS-L6 following chronic intraperitoneal injections, 5 days per week, for 4 weeks, at increasing concentrations from 10 mg/kg to 50 mg/kg. Mice showed no signs of toxicity under any of these conditions. LC/MS analysis performed in parallel allowed the detection of the molecule in the sera of animals, which correlated with the injected amounts (Fig. 6A, left).

To evaluate the effect of MS-L6 treatment on tumor growth, subcutaneous xenograft cell models (CDX) representative of the 2 non-Hodgkin B lymphoma subtypes were established (Fig. 6A, right). Since the K422 cell line failed to generate tumors after xenotransplantation despite several attempts, the SUDHL4 cell line was chosen. Remarkably, MS-L6 treatment induced a significant reduction in the volume of RL tumors and blocked the growth of SUDHL4 tumors, providing evidence for the antitumor properties of the MS-L6 molecule in these preclinical models (Fig. 6B). These observations are consistent with the cytotoxicity data observed in vitro.

## Discussion

Given the well-established role of mitochondrial respiration in tumorigenesis, there is a growing interest in using OXPHOS inhibitors for cancer treatment. MS-L6 has unique properties since it does not accumulate in the mitochondria but induces a dual effect on the MRC and exerts antitumor activity in mouse preclinical models.

Highly potent ETC-I inhibitors may exert adverse effects in clinical use. Phenformin was withdrawn from the market due to an increased risk of lactic acidosis [37]. Recently, a phase 1 clinical trial of IACS-01759 showed toxic levels of blood lactate and peripheral neuropathy [25]. Clinical trial discontinued due to limited antitumor activity at lower doses. Thus, there is an urgent need to develop new OXPHOS inhibitors that are not toxic in mice or humans. IM-156, a metformin analog, demonstrated a favorable safety profile and tolerability at the recommended Phase 2 dose [26]. MitoTAM has also successfully completed Phase I clinical trials. It belongs to a new class of anticancer agents tagged with a (TPP + ) cation. MitoTAM [19] is a tamoxifen derivative that both inhibits ETC-1 and uncouples OXPHOS allowing specific targeting of senescent cells [38]. Like MitoTAM, MS-L6 couples these two effects. However, unlike MitoTAM it does not accumulate in the mitochondria.

ETC-1 consists of 45 protein subunits organized into four functional modules that provide both electron transfer and proton pumping [39]. By targeting one or more of these proteins, it is conceivable that MS-L6 cumulates two effects. MS-L6 probably binds to the ubiquinone site, explaining its inhibitory properties in electron transfer. The uncoupling mechanisms of MS-L6 remains unknown and a non-specific protonophore activity cannot be excluded.

MS-L6 behaves as a stronger ETC-I inhibitor than as an uncoupler. ΔΨm is determined by the balance between the processes that generate and consume the proton gradient to regulate OXPHOS and ATP yield. When mitochondria are energized with ETC-II substrates, MS-L6 results in small drop in ΔΨm as the MRC compensates for proton re-entry. Conversely, when mitochondria are energized with ETC-I substrates, MS-L6 induces a strong ΔΨm drop due to inhibition of ETC-I, as the MRC cannot compensate for proton re-entry in this case. The same behavior was observed at very low doses of FCCP when ETC-I was inhibited by rotenone in the absence or presence of ETC-II substrate (Fig. S2D). NDI1 complementation assays demonstrate that inhibition of ETC-I is the key mechanism underlying MS-L6’s antiproliferative effects. NDI1 is not a proton pump and it only catalyzes electron transfer from NADH to the ubiquinone pool, without pumping protons from the matrix to the IMS [40]. However, by bypassing inhibition of ETC-I, the expression of NDI-1 allows the downstream complexes to function. Accordingly, NDI-1 almost completely restores cell growth in the presence of IACS-010759 or rotenone, but only partially with MS-L6 because the uncoupling activity is not compensated.

The double stress induced by MS-L6 requires double compensation, which may contribute to its antitumor properties. ETC-I inhibition forces cells to rely on “aerobic glycolysis” while uncoupling consumes much of the ATP generated by aerobic glycolysis. Cancer cells can survive ETC-I inhibition if they are able to maintain ΔΨm [3] by reversing the activity of ATP synthase. In addition to its effect on energy status, which explains its cytostatic activity, MS-L6 is also cytotoxic in some but not all the cell lines tested. Our results support the hypothesis that cell death is due to MS-L6-induced ROS production. Why MS-L6 induces ROS production in only some cell lines will deserve to be investigated.

Although an in vitro toxic concentration of 10 µM–50 µM is considered a negative criterion for a molecule to be developed as a drug candidate, MS-L6 shows antitumor efficacy in the preclinical mouse models tested, with no apparent toxicity upon chronic treatment. The toxicity of MS-L6 on human PBMCs is either comparable or potentially even lower than that observed with IACS-010759. Various uncouplers are more effective against cancer cells than normal cells [41]. Several of these compounds are currently in clinical trials for cancer treatment, although none have been approved for clinical use [41]. The uncoupling properties of MS-L6 may partially explain its good in vivo tolerability and is supposed to potentiate its antitumor properties. In addition, the metabolic stress induced in tumor cells may serve as an initiator of a pro-inflammatory response that sustains its antitumoral efficacy [42].

Optimizing the antitumor efficacy of MS-L6 may require a combination with inhibitors interfering with metabolic pathways activated in tumor cells but also compounds targeting stromal and immune cells of within the microenvironment. This approach merits to be explored.

Taken together, our results suggest that MS-L6 or its structural analogues may be promising new chemotherapeutic agents.

## Materials and methods

### Chemical compounds

MS-L6 was previously described [30, 31] and provided by CERMN (Caen, France). The chemical compounds and kit assays are described in the Supplementary Material and Methods section.

### Cell culture

The two human non-Hodgkin’s B-cell lymphoma cancer cell lines, RL (CVCL_1660) and Karpas422 hereafter referred to as K422 (CVCL_1325), were grown in RPMI 1640 medium GlutaMAX™ supplemented with 10% fetal bovine serum (FBS) and 1% penicillin-streptomycin. All other human cell lines are described the Supplementary Material and Methods file and were maintained under the same culture conditions. Cell line authentication was performed using PCR assays targeting short tandem repeat (STR) markers in DNA as fingerprints. The absence of mycoplasma was determined routinely.

Hepatocytes were isolated from Wistar rats according to the method of Berry and Friend [43] and modified by Groen et al. [44]. Hepatocytes were seeded on PB-60 dishes in a mixture containing 66% DMEM 4.5 g/L glucose (Pan Biotech) and 22% M199 medium supplemented with 10% FBS, 2 mM glutamine, 1% penicillin-streptomycin and 0.2 mg/mL fatty acid-free bovine serum albumin (BSA-FAF, Dutscher). Cells were maintained in a humidified incubator (37 ^o^C–5% CO_2_).

#### Generation of NDI-overexpressing cells

Plasmids pLV[Exp]-EGFP:T2A:Puro-EF1A>sce_NDI1, pLV[Exp]-EGFP:T2A:Puro-EF1A>sce_mCherry and titered lentiviral particles were purchased from VectorBuilder. K422 lymphoma cells were transduced using a spinoculation protocol. Briefly, 1 × 10^6^ cells were resuspended in fresh RPMI medium in a 6-well plate before adding 3 MOI of p_NDI1 or p_mCherry lentiviral particles per well. Plates were centrifuged at 1000 *g* for 1 h at 32 °C, then incubated at 37 °C/5%CO_2_. On day 3 after transduction, 1 µg/mL puromycin was added to the medium to select only the transduced cells.

### Isolation of mitochondria from rat liver and preparation of sub-mitochondrial particles

Mitochondria were isolated from Wistar rat livers through differential centrifugation in a medium containing 250 mM sucrose, 25 mM Tris-HCl and 1 mM EGTA as previously described [45]. Sub-mitochondrial particles were prepared by disruption of mitochondrial membranes by exposing isolated mitochondria to repeated freeze/thaw cycles followed by incubation in ultrapure water (hypoosmotic shock). Mitochondrial disruption was demonstrated by the large increase in oxygen consumption upon addition of NADH to sub-mitochondrial particles.

### Measurement of Oxygen Consumption Rate (OCR)

OCR was measured using the MT200 respirometer cell (Strathkelvin Instruments): a thermostatically controlled respiratory chamber equipped with a Clark oxygen electrode and a magnetic stirrer to ensure continuous mixing of all components. All measurements were performed at 30 ^o^C.

#### Measurement of OCR of intact and permeabilized cells

To determine the immediate effect of MS-L6 on cellular respiration, the OCR of 5 × 10^6^ RL, 5 × 10^6^ K422 and 0.5 × 10^6^ freshly isolated rat hepatocytes was measured after titration with increasing concentrations of MS-L6. These cells were either suspended in 500 µL of complete RPMI medium for intact cell OCR measurements or in 500 µL of mitochondrial assay buffer (125 mM KCl, 1 mM EGTA, 20 mM Tris, pH 7.4) for permeabilized cell OCR measurements. In the first step, cell suspensions were placed in the respirometry chamber. For OCR measurements in intact cells, the respirometry chamber was sealed with a cap and OCR recording started immediately. To permeabilize the cells, 0.4 µg/mL digitonin was added to the cell suspensions in the respiratory chamber. Permeabilized cells were then supplied with either ETC-I-I (5 mM malate, 2.5 mM glutamate) or ETC-II (5 mM succinate) substrates. In the case of permeabilized cells energized with succinate, 1 µM rotenone (ETC-I inhibitor) was added to evaluate OCR downstream of ETC-I. In permeabilized cells, ADP phosphorylation and resting states were stimulated by direct addition of 5 mM ADP and 1 µM oligomycin (ATP synthase inhibitor), respectively, onto the respiratory chamber. Both intact and permeabilized cells were titrated with MS-L6 by injecting MS-L6 directly onto the chamber using a Hamilton syringe.

#### Measurement of OCR in intact mitochondria and submitochondrial particles

Intact mitochondria freshly isolated from rat liver (0.5 mg/mL) were added to the respiratory chamber containing 500 µL mitochondrial assay buffer, 1 mM inorganic phosphate, ETC-I substrates (5 mM malate, 2.5 mM glutamate) and 5 mM ADP. The respiratory chamber was then sealed with a cap and titration of mitochondria with increasing concentration of MS-L6 was initiated. To determine the effect of MS-L6 on the OCR of rat liver submitochondrial particles (0.5 mg/mL) were added to the respiratory chamber containing 500 µL ultrapure water, 5 mM inorganic phosphate and ETC-I substrate (1 mM NADH). The respiratory chamber was sealed with a cap and titration of submitochondrial particles with increasing concentration of MS-L6 was initiated. MS-L6 was always added by direct injection into the chamber using a Hamilton syringe. The activity of ETC-I, -II and -IV was assessed by measuring the OCR of 0.5 mg/mL submitochondrial particles suspended in 500 µL water in the presence of 5 mM inorganic phosphate, which was induced by 1 mM NADH, 2 mM succinate and 250 µM/100 µM TMPD/ascorbate, respectively, before and after the addition of 50 µM MS-L6. To determine the stability of MS-L6 in medium of cultured cells, 1 × 10^6^ of hepatocytes, RL and K422 cells were cultured in 1 mL complete medium in the presence of either DMSO (vehicle control) or 50 µM MS-L6 for up to two days. Culture media were collected at various time points of incubation and stored at −80 ^o^C until analysis. The collected culture medium was first placed in the respiratory chamber along with 5 mM inorganic phosphate and 0.5 mg/mL submitochondrial particles. The respiratory chamber was then sealed, and the OCR of submitochondrial particles was monitored before and after injection of 1 mM NADH onto the chamber using Hamilton syringe. NADH-driven OCR was determined by subtracting the OCR following NADH addition from OCR before NADH addition. All results are expressed as NADH-driven OCR in the presence of medium collected from 50 µM MS-L6-treated cells relative to that collected from DMSO-treated cells. Freshly isolated rat liver mitochondria or submitochondrial particles were also used to measure the yield of ATP synthesis and the effect of BSA on rotenone activity, respectively.

### Measurement of glucose and lactate concentrations

Glucose and lactate concentrations in the culture medium of cultured cells were measured using a Flex2 automated cell culture analyzer (Nova Biomedical). Initially, 1 × 10^6^ hepatocytes, RL and K422 cells were grown in 1 mL complete medium in 12-well plates and treated with either DMSO (vehicle control) or 50 µM MS-L6 for up to 48 h. After 24 and 48 h of culture, cells were counted and centrifuged. Supernatants were collected and stored at −80 ^o^C until analysis by Flex 2. Glucose consumption and lactate production in these cells during the first 24 h of culture were subtracted from the glucose and lactate concentrations in the culture medium, respectively, and expressed in µmol per living cell.

### Quantification of total cellular adenosine nucleotides

Intracellular levels of adenosine nucleotides (ATP, ADP and AMP) were quantified by high-performance liquid chromatography (HPLC Varian model 410). Initially, 2 × 10^6^ hepatocytes, RL and K422 cells were grown in 2 mL of complete medium in 6-well plates and then treated with either DMSO (vehicle control) or 50 µM MS-L6 for 1 or 2 days. For adenosine nucleotide extraction, cells were first washed two to three times with PBS and then lysed with 500–1000 µL of 2.5% PCA (perchloric acid) - 6.25 mM EDTA (ethylene diaminetetraacetic acid). The acidic extract was vortexed and then neutralized with 100-200 µL 2 N KOH (potassium hydroxide) - 0.3 M MOPS. The neutralized solution was centrifuged at 12,000 g for 5 minutes at 4 ^o^C. The final extract was stored at -80^o^C until later analysis by HPLC. For HPLC analysis, 75 µL of the final extract was mixed with 35 µL of mobile phase and 15 µL of 1 N HCl, and the mixture was vortexed and transferred to HPLC vials. The vials were then placed in the autosampler tray and HPLC runs were initiated (column: Polaris 5 C18-A, mobile phase: 28 mM sodium pyrophosphate decahydrate, run time: 30 min). HPLC results were provided as chromatograms showing three peaks (ATP, ADP and AMP) with different retention times. These peaks were integrated and calibrated using offline HPLC software.

### Measurement of mitochondrial membrane potential

The ΔΨm of mitochondria isolated from rat liver was assessed using the mitochondrial probe, rhodamine 123 (Thermo Fisher Scientific, R302). The fluorescence of rhodamine 123 was monitored over time using a PTI Quantamaster C61 spectrofluorometer (excitation: 498 nM; emission: 524 nM). Briefly, 2 mL of buffer containing 5 mM inorganic phosphate, 0.15% BSA, ETC-I substrates (5 mM malate, 2.5 mM glutamate) and 50 nM rhodamine 123 were initially added to the cuvette. The resulting basal fluorescence signal was stabilized for approximately one minute. A new steady state directly related to ΔΨm was reached one to two minutes after the addition of 0.5 mg/mL of freshly isolated rat liver mitochondria. This was followed by the addition of either 50 µM MS-L6 or 1 µM rotenone. Finally, FCCP was added to fully depolarize ΔΨm. Throughout the experiment, the mixture was stirred continuously with a magnetic rod. Changes in rhodamine 123 fluorescence were recorded and directly correlated with changes in ΔΨm. ΔΨm of intact RL and K422 cells was assessed by labelling cells with the two vital mitochondrial probes, tetramethylrhodamine methyl ester TMRM (Thermo Fisher Scientific, I34361) to assess ΔΨm and Mito Tracker Green (MTG) to assess mitochondrial mass. Cell suspensions were divided into two halves; the first half was incubated with 200 nM TMRM and the second half was incubated with 200 nM MTG for 20 minutes at 37 ^o^C. This was followed by treatment of each half with either DMSO (vehicle control), 50 µM MS-L6 or 1 µM rotenone for another 60 min. The TMRM labelled cell suspension was then further divided into two (one half was treated with CCCP for 5 min) and the fluorescence of TMRM, TMRM + CCCP and MTG labelled cells was measured by flow cytometry. Results were expressed as TMRM fluorescence (TMRM – TMRM + CCCP) normalized to mitochondrial mass (MTG).

### Assessment of NADH oxidizing activity of mitochondrial complex I

ETC-I activity (NADH oxidation) of rat liver submitochondrial particles was assessed spectrophotometrically by following the NADH absorbance at 340 nM. Briefly, 0.5 mg/mL submitochondrial particles were first added to 2 mL of water containing 10 mM inorganic phosphate and 1 mM KCN, which blocks electron transfer by mitochondrial ETC-IV. The recording of NADH absorbance was started immediately after the addition of NADH. This was followed by the addition of decylubiquinone as the final acceptor of electrons resulting from NADH oxidation by ETC-I. Finally, where indicated, DMSO (vehicle control) or 50 µM of MS-L6 was added. The activity of NDI (ETC-I subunit) of yeast mitochondria (*Saccharomyses cerevisiae*) was also evaluated in the same manner. Yeast mitochondria were a kindly provided by Dr. Anne Devin (IBCG CNRS, Bordeaux, France).

### Assessment of cellular proliferation

Cell proliferation was assessed by counting cells using trypan blue. Initially, 1 × 10^6^ hepatocytes, RL and K422 cells were seeded in 1 mL complete medium in 12-well plates and treated with either DMSO (vehicle control) or 50 µM MS-L6 for 48 h. At various time points, 20 µL of cell suspension was collected and mixed with 20 µL trypan blue (1:1). Cells were counted using the Luna automated cell counter (Logos Biosystem) and live cell counts were used to construct representative proliferation curves.

### Assessment of cell viability

Initially, 1 × 10^5^ RL and K422 cells were seeded in 100 µL complete medium in 96-well plates and treated with either DMSO (vehicle control) or indicated concentrations of MS-L6 or other inhibitors for 48 h. Cell viability was then assessed by either flow cytometry or biochemical assays as indicated in the text. For flow cytometric analysis, the Annexin V/propidium iodide double staining method was used. Cell suspensions were collected. Pellets were suspended in annexin-containing buffer, according to standard protocols. Propidium iodide was added to each sample immediately before analysis. Flow cytometry data acquisition was performed using BD LSRFortessa™ or automated Attune™ Flow cytometer (Thermo Fischer Scientific) which analysed all cells in a well during large-scale analysis of hematological cell lines. Data were analyzed using FlowJo software (V10, TreeStar Inc, Ashland, USA). The percentage of the population that was negative for annexin V and propidium iodide labelling was considered viable. The same experimental design was used to test the effect of MS-L6 on the large panel of hematological cell lines. Complementary experiments were performed using the CellTiter*-*Fluor™ Cell Viability Assay (Promega), a non-lytic assay that measures the relative number of viable cells based on cellular protease activity. A similar experimental design as that described above was used to plate cells and administer treatments, except that the endpoint analysis of cell viability was performed using a single fluorescent reagent added in each well after 48 h of treatment, and relative fluorescence was measured using a Fluorimeter plate reader (TECAN) according to manufacturer’s instructions.

Finally, the simultaneous determination of live and dead cells by imaging was performed using LIVE/DEAD Viability/Cytotoxicity Assay Kit (L3224 Invitrogen) and is described in the Supplementary Materials and Methods section.

### Evaluation of superoxide production analysis by flow cytometry

Cell suspensions were collected and centrifuged at 2000 rpm for 5 min. Pellets were resuspended in 1 ml culture medium containing 1 µM Mitosox probe (Life Technologies, ThermoFisher Scientific, USA) for 20 min in a humidified atmosphere with 5% CO_2_ at 37 °C, protected from light, and then immediately analyzed by FACS (BD LSR FORTESSA, Becton Dickinson, Le Pont-de-Claix, France). Laser excitation was at 532 nm for MitoSox. Fluorescence emission was collected using a 585/15 nm bandpass filter. A negative control (autofluorescence) was determined with unlabeled cells, and positive control was established after prior incubation of cells with 250 mM of tBH (Tert-butyl hydroxyperoxide, Sigma-Aldrich, France) for 20 min. Superoxide production was expressed as the percentage of positive cells above the threshold determined by autofluorescence.

### Flow cytometry immunophenotyping

#### PBMC staining and data collection

PBMC samples used for flow cytometry analysis were obtained from blood collections from the Center for Biological Resources of Lyon (BRIF: BB-0033-00046). PBMC were harvested and washed in PBS 1X. The cells were then surface stained with an antibody cocktail in Brilliant Stain buffer (BD Biosciences) for 15 min at 4 °C (Table S1 in Supplementary Data). The cells were then washed and stained with Annexin V in Annexin buffer for 10 min at room temperature. After further washing, the stained cells were acquired using a Cytek Aurora spectral flow cytometer.

#### Bioinformatical analysis of flow cytometry

For multiparametric analyses, dimension reduction and clustering were performed using OMIQ software from Dotmatics (www.omiq.ai, www.dotmatics.com). After pre-cleaning the data of aggregates, dead cells and debris, all CD45-positive and Annexin V-negative events from all samples were selected for subsequent analysis on the OMIQ platform. An op-tsne analysis was then performed to visualize the different subsets [34]). FlowSOM was run to cluster the data using meta-clustering with *k* = 50. After FlowSOM analysis, the metaclusters were grouped into commonly recognized biological populations. All clusters were plotted on traditional dot plots for phenotype confirmation as for the standard manual gating analysis.

### Preclinical evaluation of MS-L6 antitumor activity in mouse models

#### MS-L6 detection in mouse serum

All animal experiments were approved by the Animal Protection Ethics Committee of the French Ministry of Higher Education and Research under the reference APAFIS#2016083009597532. We confirm that all experiments were performed in accordance with the relevant guidelines and regulations of this committee.

A preliminary determination of the maximum tolerated dose (MTD) of MS-L6 to be used for preclinical evaluation of its antitumor efficacy was first performed. In these experiments, submandibular blood samples were collected 1 h after intraperitoneal injections of 10–50 mg/kg MS-L6. Plasma was stored at −20 °C until analysis. The chromatographic system used for MS-L6 quantification consisted of an Ultimate 3000 system coupled to an MS/HRMS Q-Exactive Plus Orbitrap mass spectrometer (Thermo Scientific, Germany) equipped with electrospray ionization source (LC-MS/HRMS). The detailed protocol is described in the Supplementary Materials and Methods section.

#### Evaluation the antitumor efficacy of MS-L6 in cell lines derived xenografts (CDX) models

Experiments were performed by ANTINEO (Lyon, France), a CRO specialized in preclinical oncology. The detailed protocol is described in the Supplementary Materials and Methods section. Briefly, 5 × 10^6^ RL or SUDHL4 cells per injection were first injected subcutaneously into SCID mice. Mice were randomized when tumors reached a mean volume of 100 mM^3^ for the 2 groups (control and MS-L6). Tumor volume was measured three times per week. MS-L6 was administered by intraperitoneal injection five times a week, at a dose of 50 mg/kg. Control diluent (DMSO) was also administered by intraperitoneal injection five times per week.

### Statistical analysis

Statistical significance was determined using the GraphPad Prism 9 software package. Unless mentioned, the t-test was used to assess the significance of differences between control and experimental conditions, with paired tests for within-group comparisons and unpaired tests for between-group comparisons. IC50 values were determined by fitting dose-response curves. Vmax and Km were determined by constructing Michaelis-Menten curves. The two-way ANOVA test followed by Dunnet’s multiple comparison test was used in case of flow cytometric immunotyping experiments. For in vivo experiments, the non-parametric Mann-Whitney test was performed on tumour volumes (mm3) measured on the day of euthanasia. *P* values < 0.05 are considered significant and depicted as follows: *P ≤ 0.05; **P ≤ 0.01; ***P ≤ 0.001; ****P ≤ 0.0001.

### Supplementary information

SUPPLEMENTAL MATERIAL

## Data Availability

All data generated or analyzed during this study are available from the corresponding author on reasonable request.
