# Supplementary material for: A novel inhibitor of the mitochondrial respiratory complex I with uncoupling properties exerts potent antitumor activity
Source: Cell Death Dis. 2024 May 2;15(5):311. doi: 10.1038/s41419-024-06668-9 (PMC11065874; doi:10.1038/s41419-024-06668-9)
Supplement: Supplementary file 1 — SUPPLEMENTAL MATERIAL [file 41419_2024_6668_MOESM1_ESM.pdf]

SUPPLEMENTARY INFORMATIONS

A novel inhibitor of the mitochondrial respiratory complex I with uncoupling properties exerts potent antitumor activity.

Alaa Al Assi[1], Solène Posty[2], Frédéric Lamarche[1], Amel Chebel[3], Jérôme Guitton[4], Cécile Cottet-Rousselle[1], Renaud Prudent[5], Laurence Lafanechère[5], Stéphane Giraud[6], Patrick Dallemagne[7], Peggy Suzanne[7], Aurélie Verney[3], Laurent Genestier[3], Marie Castets[2], Eric Fontaine[1]*, Marc Billaud[2]* and Martine Cordier-Bussat[2]*

Running title: A novel OXPHOS inhibitor with uncoupling properties

Conflict of interest statement: The authors declare no competing interests.

A

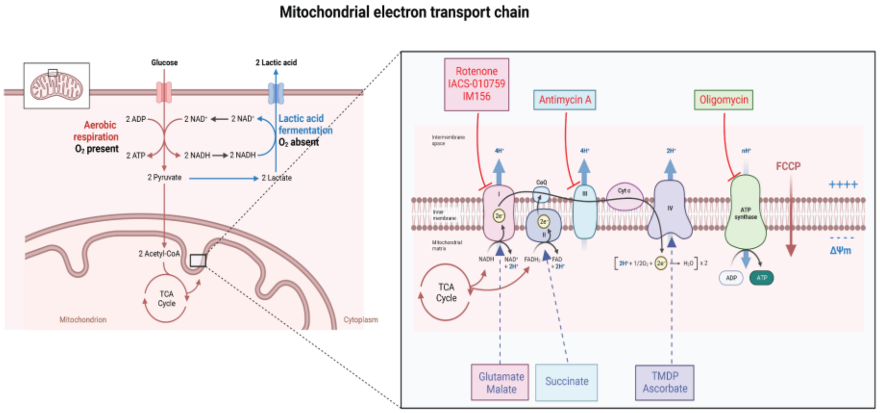

Figure S1: Schematic diagram of mitochondrial respiratory chain and inhibitors.
This figure reports the main chemical steps of OXPHOS, including the four protein complexes of the electron transport chain (ETC) and ATP synthase, with classical substrates and inhibitors used in the experiments. Briefly, the respiratory chain can be fed by electrons coming from NADH (equivalent to Glutamate/Malate), succinate or TMPD/Ascorbate (artificial electron donor) through ETC-I, ETC-II and ETC-IV, respectively. These electrons are then transferred to ETC-IV where they are accepted by O_2, the ultimate electron acceptor. The forward electron flux through the ETC drives proton pumping, resulting in a proton gradient across the IMM. Finally, ATP synthase exploits the energy of this gradient to drive the phosphorylation of ADP into ATP.
The chemical structures of ETC-1 inhibitors used in these study is indicated in figure S1B

B

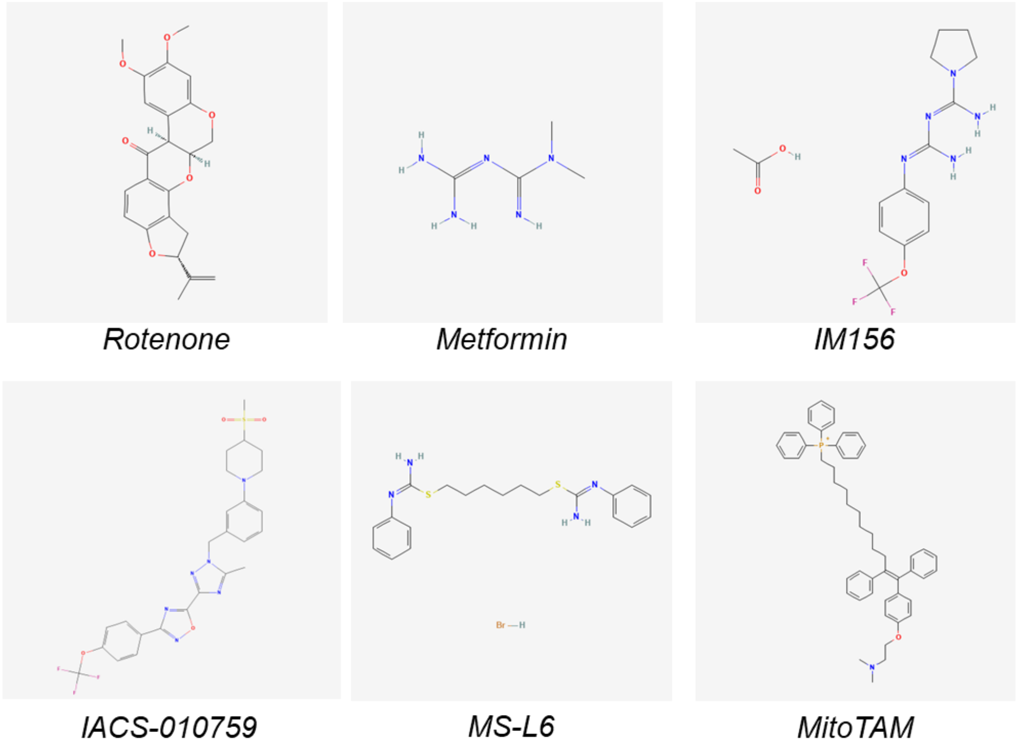

S2A: Interaction/competition between MS-L6 and DecylUbiquinone.

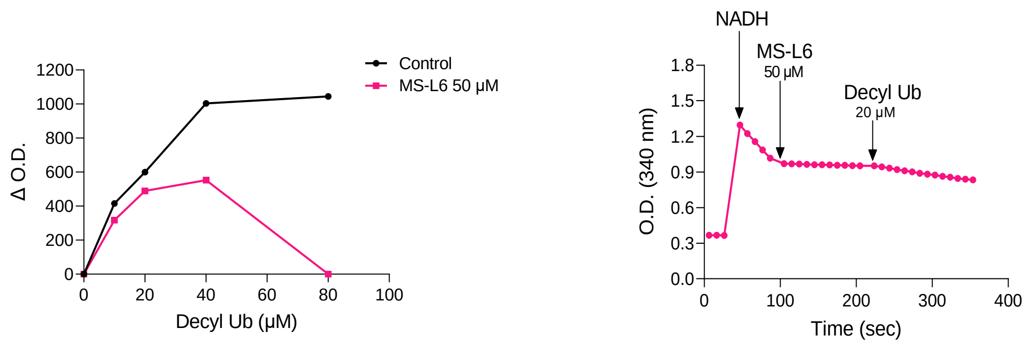

S2B : BSA does not affect Rotenone activity

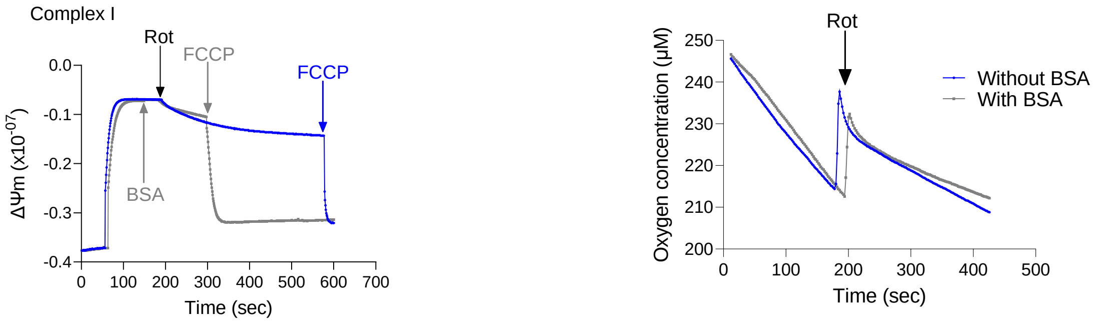

S2C: Quantification of MS-L6 uncoupling effect

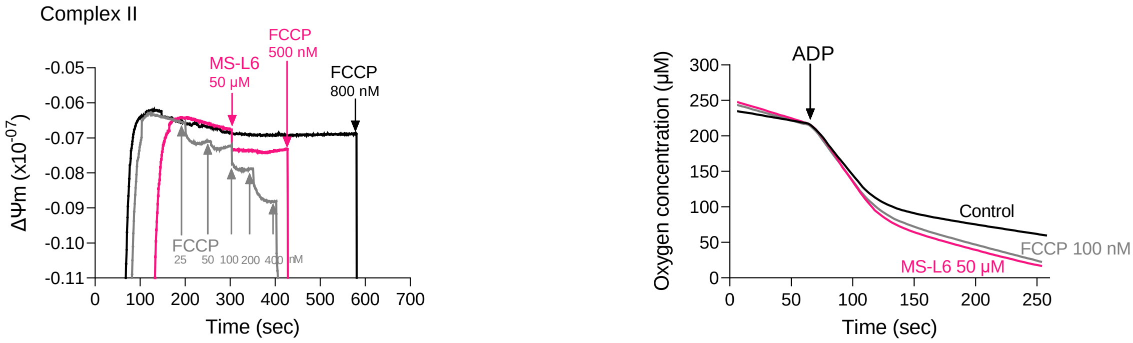

S2D: For the same decoupler concentration, the depolarizing effect depends on the Vmax of the respiratory chain.

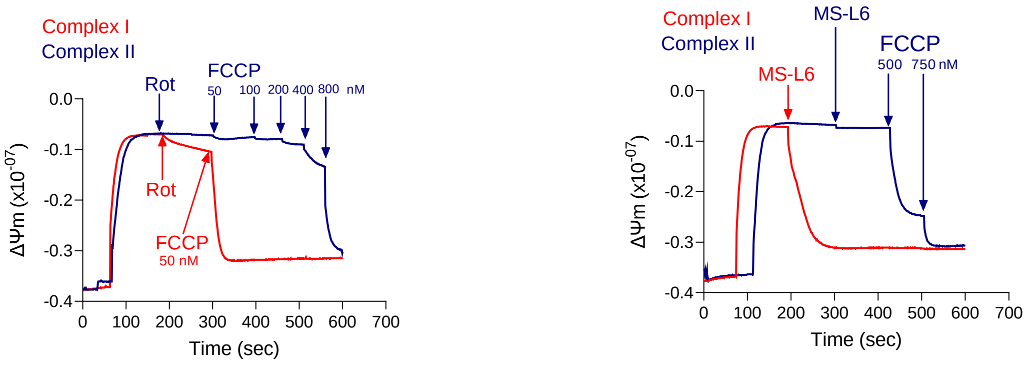

S3A:

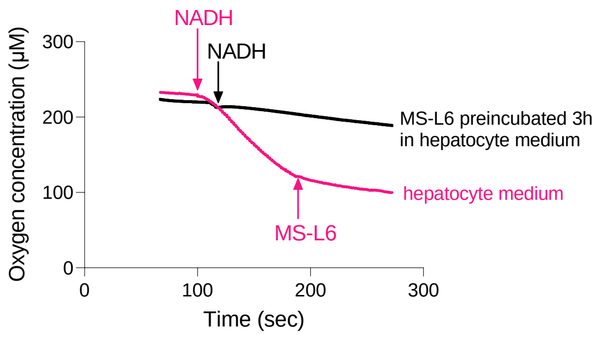
NADH
NADH
MS-L6 preincubated 3h
in hepatocyte medium
hepatocyte medium
MS-L6
Oxygen concentration (µM)
Time (sec)

S3B:

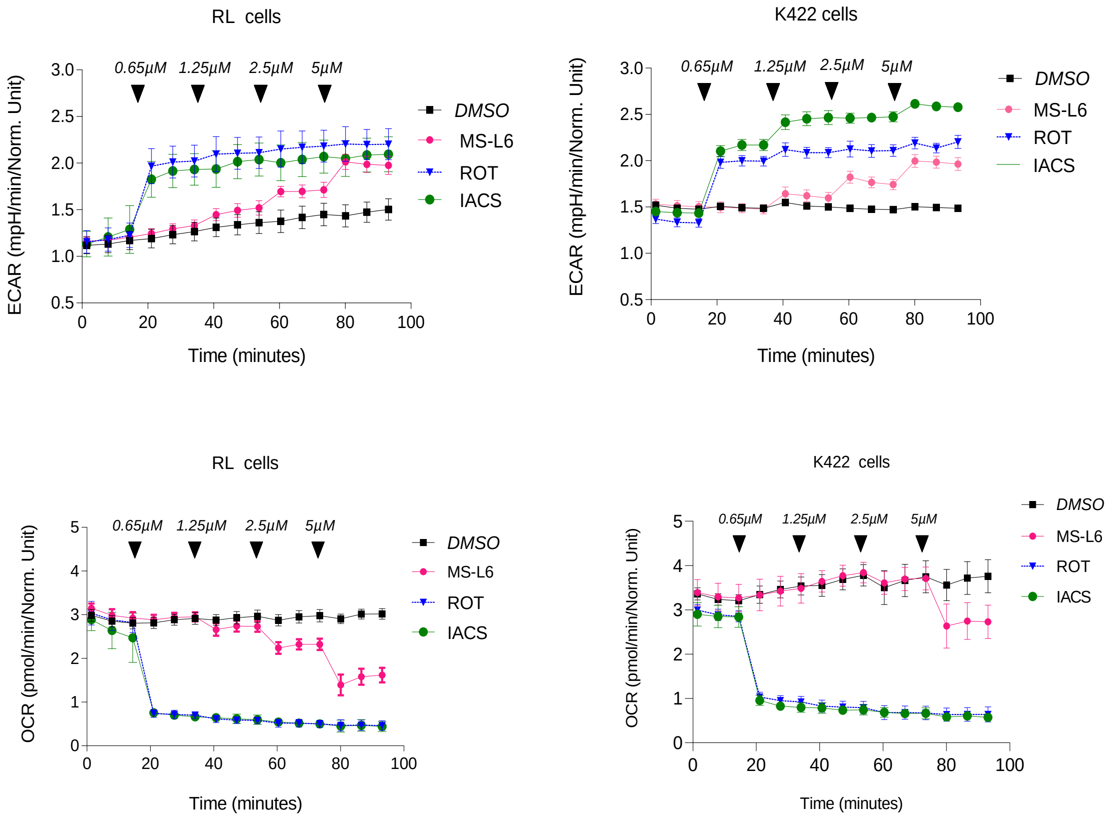
RL cells
K422 cells
0.65µM
1.25µM
2.5µM
5µM
DMSO
MS-L6
ROT
IACS
ECAR (mpH/min/Norm. Unit)
OCR (pmol/min/Norm. Unit)
Time (minutes)

Figure S4

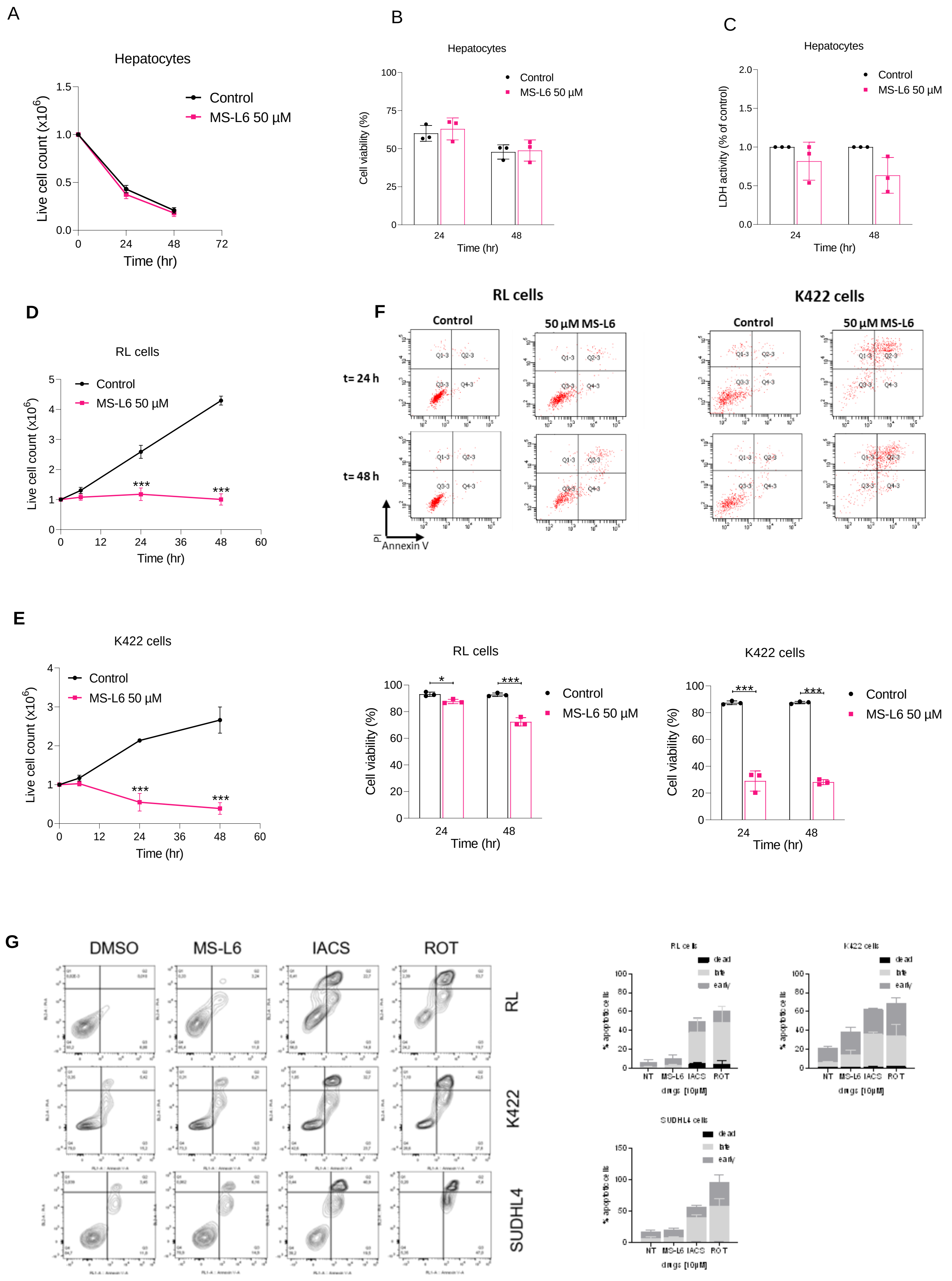
A
Hepatocytes
Control
MS-L6 50 µM
Live cell count (x10^6)
Time (hr)
B
Hepatocytes
Control
MS-L6 50 µM
Cell viability (%)
Time (hr)
C
Hepatocytes
Control
MS-L6 50 µM
LDH activity (% of control)
Time (hr)
D
RL cells
Control
MS-L6 50 µM
Live cell count (x10^6)
Time (hr)
***
F
RL cells
K422 cells
Control
50 µM MS-L6
t= 24 h
t= 48 h
PI
Annexin V
Q1-3
Q2-3
Q3-3
Q4-3
E
K422 cells
Control
MS-L6 50 µM
Live cell count (x10^6)
Time (hr)
***
RL cells
K422 cells
Cell viability (%)
Control
MS-L6 50 µM
Time (hr)
*
***
G
DMSO
MS-L6
IACS
ROT
RL
K422
SUDHL4
RL cells
K422 cells
SUDHL4 cells
dead
late
early
% apoptotic cells
NT
MS-L6
IACS
ROT
drugs [10µM]

Figure S5

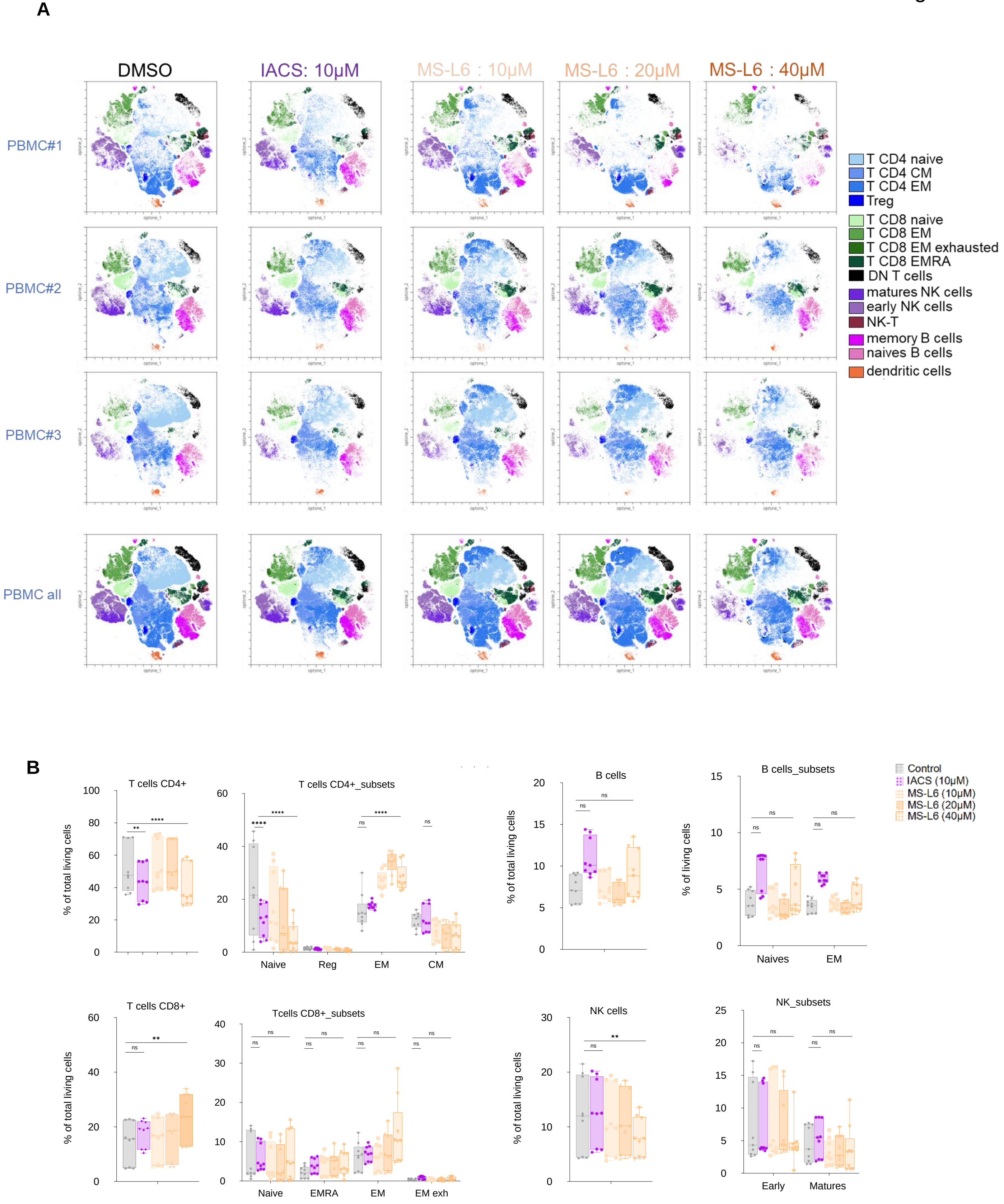
A
DMSO
IACS: 10µM
MS-L6 : 10µM
MS-L6 : 20µM
MS-L6 : 40µM
PBMC#1
PBMC#2
PBMC#3
PBMC all
T CD4 naive
T CD4 CM
T CD4 EM
Treg
T CD8 naive
T CD8 EM
T CD8 EM exhausted
T CD8 EMRA
DN T cells
matures NK cells
early NK cells
NK-T
memory B cells
naives B cells
dendritic cells
B
T cells CD4+
T cells CD4+_subsets
B cells
B cells_subsets
Control
IACS (10µM)
MS-L6 (10µM)
MS-L6 (20µM)
MS-L6 (40µM)
% of total living cells
% of living cells
Naive
Reg
EM
CM
Naives
T cells CD8+
Tcells CD8+_subsets
NK cells
NK_subsets
EMRA
EM exh
Early
Matures

Figure S6

A

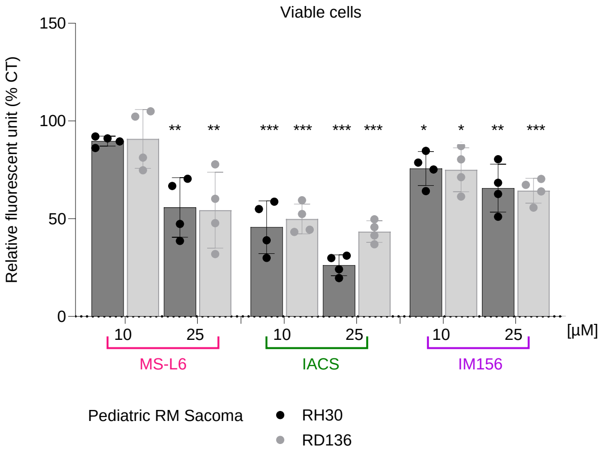

B

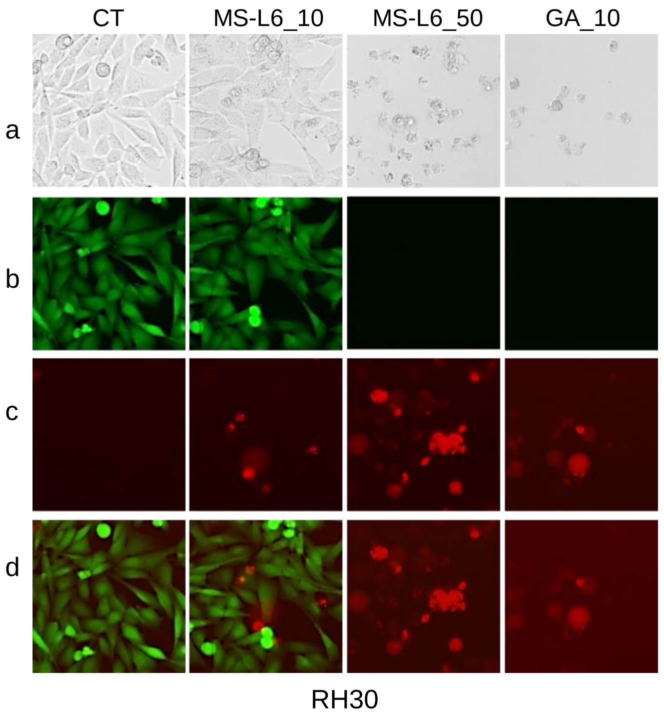

C

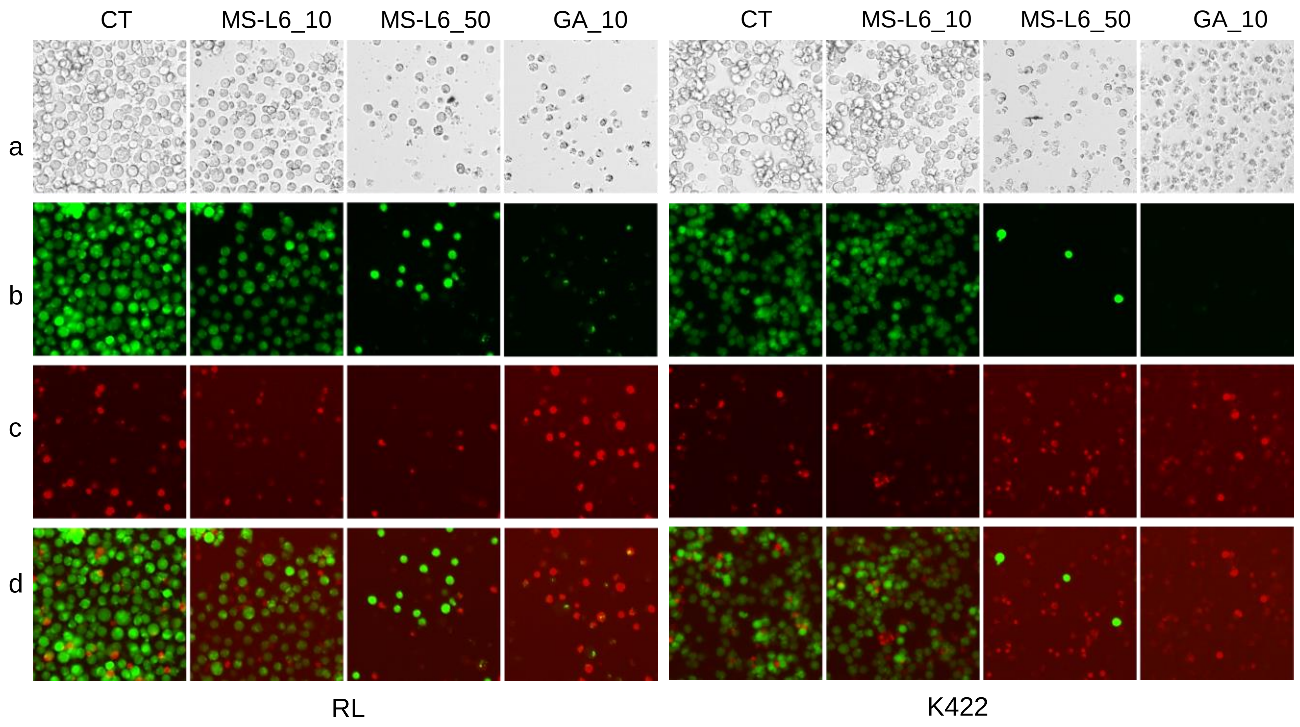

Figure S7

A

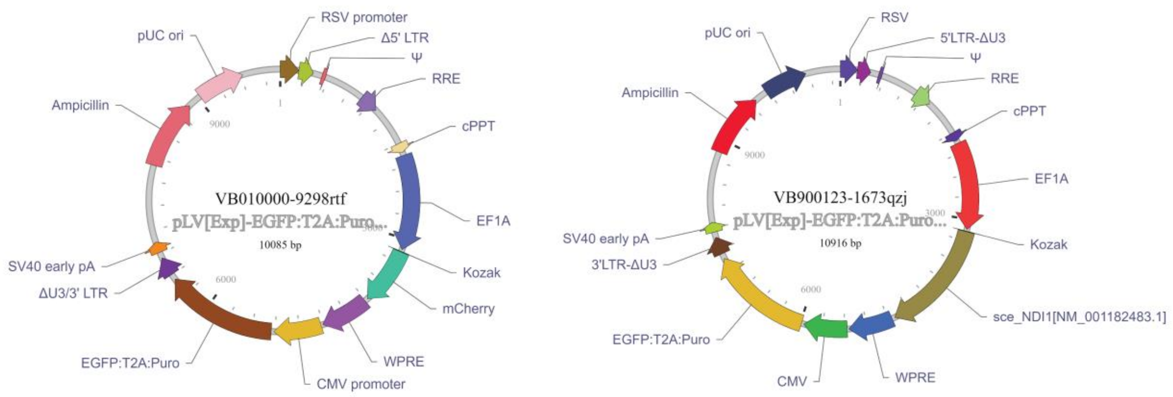

B

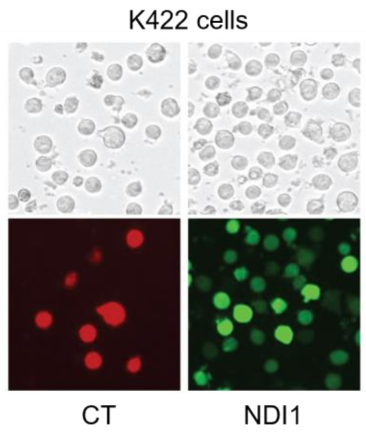

C

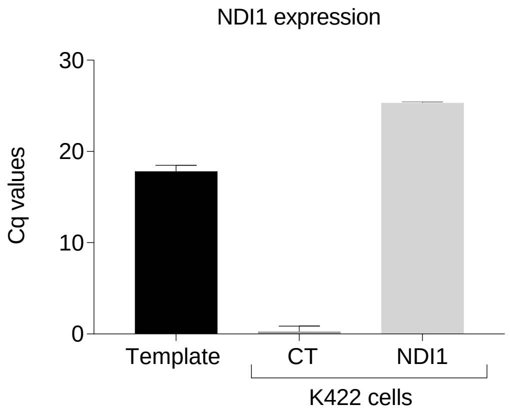

D **Cells overexpressing NDI1 are rotenone-insensitive**

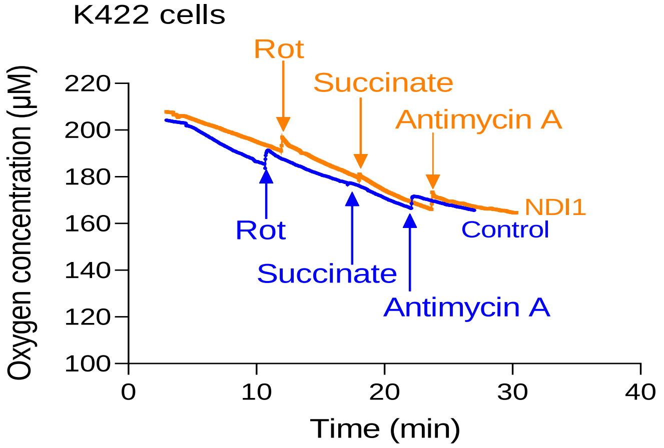

SUPPLEMENTARY FIGURE LEGENDS

Figure S1: **Schematic representation of the OXPHOS machinery and inhibitors**.

Panel (A): This figure shows the main chemical steps of OXPHOS, including the four protein complexes of the electron transport chain (ETC) and ATP synthase, with the classical substrates and inhibitors used in the experiments. Briefly, the respiratory chain can be supplied with electrons coming from NADH (equivalent to glutamate/malate), succinate or TMPD/ascorbate (artificial electron donor) via ETC-I, ETC-II and ETC-IV, respectively. These electrons are then transferred to ETC-IV where they are accepted by O_2, the ultimate electron acceptor. The forward electron flux through the ETC drives proton pumping, resulting in a proton gradient across the IMM. Finally, ATP synthase uses the energy of this gradient to drive the phosphorylation of ADP to ATP.

Panel (B): presented the structure of the MS-L6 and other ETC-1 inhibitors.

Figure S2: Uncoupling effect of MS-L6

***S2A**: Interaction/competition between MS-L6 and decylubiquinone.*

Left panel shows ETC-I activity measured by following NADH absorbance spectrophotometric assay at 340nM of rat liver sub-mitochondrial particles in the presence of DMSO (vehicle control) or 50µM MS-L6. Sub-mitochondrial particles were first incubated with 1 mM NADH in the presence of 1mM KCN, and then increasing concentrations of decylubiquinone were added. Decylubiquinone did not inhibit ETC-I activity under control condition but higher concentrations of decylubiquinone tended to inhibit NADH oxidation, especially in the presence of 50 µM MS-L6(Fig.S2A). However, the relief of MS-L6 inhibition of NADH oxidation was observed at 50 µM MS-L6 in the presence of 20 µM decylubiquinone (Fig.S2B)

***S2B**: BSA has no effect rotenone activity.*

Left panel shows ΔΨm of 0.5mg/mL of intact rat liver mitochondria energized with ETC-I substrate (Glut/Mal), after treatment with 1µM rotenone in the presence (grey lane) or absence (blue lane) of

0.15% BSA. When indicated 800 nM FCCP was added to fully depolarize ΔΨm. The depolarization was slightly lower in the presence of BSA, presumably due to chelation of free fatty acids.

Right panel shows the OCR of submitochondrial particles energized with NADH in the presence or absence of 0.15% BSA. The decrease in OCR induced by rotenone was similar in both conditions.

S2C*: *Quantification of the MS-L6 uncoupling effect.

Left panel shows ΔΨm of 0.5mg/mL of intact rat liver mitochondria energized with ETC-II substrate (succinate), after treatment with 50 µM MS-L6 (blue lane) or increasing concentrations of FCCP (green lane). Control (Brown trace). MS-L6-induced depolarization was between that induced by 50nM and 100 nM FCCP.

Right panel shows oxygen consumption of mitochondria with succinate in the presence or absence of 50 µM MS-L6 or 100 nM FCCP. Where indicated 400µM ADP was added. Oxygen consumption during ATP synthesis was higher in the presence of 50 µM MS-L6 or 100 nM FCCP than in the control condition.

S2D*: *At the same uncoupler concentration, the depolarizing effect depends on the Vmax of the respiratory chain. *Panels* show ΔΨm of 0.5mg/mL of intact rat liver mitochondria energized with ETC-I substrate (Glut/Mal) or ETC-II substrate (Succ). ETC-I was then inhibited with either rotenone (left) or MS-L6 (right) followed by sequential addition of FCCP. In the presence of rotenone (left), 50 nM FCCP completely depolarized mitochondria fed with ETC-I substrate, whereas it induced a visible but weak depolarization when mitochondria were fed with ETC-II substrate (succinate). When 50 µM MS-L6 was added instead of rotenone, it completely depolarized mitochondria fed with ETC-I substrate, whereas it induced a visible but weak depolarization when mitochondria were fed ETC-II substrate (succinate).

Figure S3:

Panel A: Incubation of MS-L6 for 3 hours at 37°C in the hepatocyte culture media did not decrease MS-L6 activity. Panel A shows the OCR of submitochondrial particles incubated in culture media of hepatocytes supplemented with NADH. 50µM MS-L6 inhibits OCR when added immediately (orange

lane) or when preincubated for 3 hours at 37°C (grey lane), indicating that hepatocyte culture media did not affect MS-L6 bioavailability.

Panel B shows real time OCR and ECAR measured by Seahorse technology in RL and K422 cells treated with increasing final concentrations of MS-L6 (0.65µM to 5µM), IACS-010759 (0.65µM to 5µM) or rotenone (0.65 to 5nM). The arrows indicate the progressive addition of inhibitors to achieve the final concentration indicated for MS-L6 and IACS-01075. The final concentration is 10-fold lower for rotenone. Data are presented as mean ± SD of 8 experimental replicates, n=3; these graphs are representative of 3 independent experiments n=3.

Figure S4: MS-L6 induces cancer cells death.

Panel (A) shows real time cellular proliferation of hepatocytes treated with DMSO (vehicle control) or 50µM MS-L6. Initially, 1 x 10^6 cells were seeded, and cells were counted at different time points of incubation. Data are presented as means ± SD, n=3.

Panel (B) shows the percentage of viability of hepatocytes after flow cytometric analysis 24 and 48 hours after treatment with either DMSO (vehicle control) or 50µM MS-L6. Initially, 1 x 10^6 hepatocytes were seeded and were counted after 24 and 48 hours of incubation. Data are presented as means ± SD, n=3.

Panel (C) shows LDH activity measured in the culture medium collected from hepatocytes treated with 50µM MS-L6 compared to control cells after 24 and 48 hours of incubation. Data are presented as mean ± SD, n=3.

Panel (D-E) show real time cellular proliferation of RL and K422 cells treated with DMSO (vehicle control) or 50µM MS-L6. Initially, 1 x 10^6 cells were seeded, and cells were counted at different time points of incubation. Data are presented as means ± SD, n=3.

Panel (F) shows typical flow cytometry dot plots of RL and K422 cells co-labelled with Annexin V and PI and treated as indicated. Histograms below represent quantitative analysis

of these data showing the percentage of viability of RL and K422 cells 24 and 48 hours after treatment with either DMSO or 50µM MS-L6. Data are presented as means ± SD.

Panel (G) shows data obtained in a representative experiment of flow cytometry analysis after Annexin V/PI double staining. Analysis was performed 48h after treatment of RL, K422 and SUDHL4 cells with DMSO diluent (NT), 10µM MS-L6, 10µM IACS-0105-759 or 1µM rotenone. *Left panel* shows a typical flow cytometry contour plot obtained in parallel after Annexin V/PI double staining analysis (X axis: Annexin, Y axis: PI) in one replicate well. *Right panel* shows histograms of quantification of these analyses, presented as the percentage of cells in the different apoptosis stages of all well replicates. Data are presented as mean ± SD, and from one representative experiment n>3.

Statistical significance was determined as described in materials and methods.

Figure S5: **Analysis of MS-L6 effects on human PBMCs by flow cytometry immunophenotyping**.

Panel A: In this op-tsne graphs, each dot represents one cell, and each colour represents one subpopulation identified by labelling with the antibodies described in Table 1. The data sets collected individually for each patient (PBMC#1-3) are shown in Fig.S5A, according to the treatment applied to the cells (diluent: DMSO, IACS-010759 10µM and MS-L6 10-40µM). Computer analysis allows cumulative visualisation of all data (PBMC#all) partially presented in the figure 4. *Panel B*: shows the percentage of live cells in each population. Human CD8 naive central memory (T_{CM}), effector memory ($_{EM}$), Human CD8 effector memory cells re-expressing CD45RA (T_{EMRA}). CD4+ T cells are the most numerous, representing about 50% of all cells. Each point on boxes and whiskers (Min to Max, all points) represents the value of a single technical replicate, 3 technical replicates per each of the 3 donor samples. As the percentages are shown, it is possible to see an increase in certain sub-populations which become the majority after treatment, such as CD4/CD8+ EM T cells treated with MS-L6. At

40µM, MS-L6 treatment significantly affects CD4+ naive and CM T cells and CD8+ naïve T cells. At 40µM MS-L6, the percentage of total NK cells was significantly reduced, although there was no significant variation in the sub-populations. At 40µM MS-L6, the percentage of B cells did not differ from DMSO control samples, as shown by the variation in cell counts in Figure 4, which was not significant.

Statistical significance was determined as described in materials and methods.

Figure S6: MS-L6 induces lymphoma and paediatric sarcoma cancer cell death.

Live/dead cell analysis of B-lymphoma (RL and K422) and rhabdomyosarcoma (RH30) cells treated with 10µM and 50µM MS-L6. Treatment with 10µM gambogic acid was used as an inducer of cell death. Images observed 48h after treatment are shown: bright field (a), green filter to detect labelling of viable cells (b), red filter to detect labelling of dead cells, and merged images to detect both (d).

Figure S7: NDI1 complementation experiments.

Panel (A) shows maps of lentiviral vectors used to express yeast NDI1 protein and its corresponding control vector. *Panel (B)* The NDI1 lentiviral vector exhibits expression in green, while the control vector shows expression in red, after lentivirus transduction of K422 cells. *Panel (C)* reports NDI1 expression measured by RT-qPCR, in CT and NDI1 K422 infected cells. NDI1 DNA template was used as a positive control.

Panel (D): *Cells overexpressing NDI1 are rotenone insensitive. Panel D* reports OCR of sub-mitochondrial particles (thawed K422 cells further disrupted by osmotic choc) supplemented with NADH. Rotenone did not decrease OCR in cells overexpressing NDI1 (in fact, it slightly increased OCR). Control and NDI1-overexpressing cells remained sensitive to succinate and antimycin A.

SUPPLEMENTARY MATERIAL& METHODS

Effect of rotenone on cells overexpressing NDI1

For OCR measurement of cells overexpressing or not NDI1 frozen cells (10 million cells) were thawed and placed directly into ultrapure water supplemented with 10 mM Pi for 5 min (osmotic shock). The respiratory chamber was then sealed with a cap and 0.5 mM NADH, 1 µM rotenone, 5 mM succinate and 100µM antimycin A were added sequentially.

Effect of BSA on rotenone sensitivity

ΔΨm of rat liver mitochondria incubated in the presence of ECT-I substrate was evaluated with the mitochondrial probe, rhodamine 123 as described above. Rotenone (1µM) was added in the presence or absence of BSA (0.15%).

The effect of rotenone (1µM) was also measured by OCR in sub-mitochondrial particles (1 mg/ml) in the presence or absence of BSA (0.15%).

Quantification of the MS-L6 uncoupling effect.

ΔΨm of rat liver mitochondria incubated in the presence of ECT-II substrate was evaluated with the mitochondrial probe, rhodamine 123 as described above. Increasing concentrations of FFCP were used to progressively abolish ΔΨm and were compared to the depolarization induced by 50 µM MS-L6.

Effect of FCCP on ΔΨm when mitochondria were incubated with rotenone in the presence of ECT-I or ECT-II substrate, respectively.

ΔΨm of rat liver mitochondria incubated in the presence of ECT-I or ECT-II substrates was evaluated with the mitochondrial probe, rhodamine 123 as described above. Rotenone (1 µM)

was then added and increasing concentrations of FFCP were used to progressively abolish ΔΨm.

***Measurement of OCR and ECAR in intact cells using Seahorse technology*:**

Real-time metabolic analysis was performed using the Seahorse Bioscience XFe96 Extracellular Flux Analyzer (Agilent), which allows simultaneous measurement of cellular oxygen consumption rate (OCR in pmol/min) and extracellular acidification rate (ECAR in mpH/min). On the day of the assay, 1.5 x 10^5 RL and 2 x 10^5 K422 cells in exponential growth were seeded onto Seahorse 96-well plates coated with Corning Cell-Tak (TMsub), according to the manufacturer's instructions. The number of cells seeded was optimized to ensure 70-80% confluence. The culture medium was replaced with Seahorse XF RPMI assay medium pH 7.4 (Agilent) and the plate was pre-incubated for 30 min at 37°C in a non-CO_2 incubator. Simultaneous OCR and ECAR were then measured according to supplier's instructions under basal conditions (injection of inhibitor diluent *i.e.,* DMSO) and after sequential injections of different inhibitors (MS-L6, rotenone or IACS-010759). OCR and ECAR levels were normalized to the number of cells per well using Agilent Seahorse XF imaging and cell counting procedures.

Live/dead cell analysis

The day before treatments, cells were seeded in 80µL/well of RPMI (1% penicillin-streptomycin, 10% SVF) in a 96-well plate with a transparent bottom. Treatments were added the next day in 20µL/well at the appropriate dilution to obtain the final working concentration of each compound. After 48h of treatment, the simultaneous determination of live and dead cells by imaging was performed using the LIVE/DEAD Viability/Cytotoxicity Assay Kit (L3224 Invitrogen) according to manufacturer's protocol. This two-color fluorescence cell viability assay is based on the simultaneous detection of live and dead cells with two probes

that detect parameters of cell viability – intracellular esterase activity with green fluorescence in live cells (ex/em ~495nM/~515nM) and plasma membrane integrity – DNA with red fluorescence in dead cells (ex/em ~495nM/~635nM). Briefly, 100µl/well of a 2x working solution of 2µM calcein AM and 4µM EthD-1 was added according to manufacturer's instructions, and images were acquired using EVOS® FL Imaging System (Life Technology).

Quantification of MS-L6 in mouse sera:

The chromatographic system used for MS-L6 quantification consisted of an Ultimate 3000 system coupled to an MS/HRMS Q-Exactive Plus Orbitrap mass spectrometer (Thermo Scientific, Germany) equipped with an electrospray ionization source (LC-MS/HRMS). Chromatographic separation was performed on an Atlantis-Hilic chromatographic column (150 × 2.1mM, 3µm) (Waters, USA) using a gradient elution program. The mobile phase consisted of water, acetonitrile and acetate buffer (100mM, pH 5.0). Data acquisition was performed in full scan mode with mass resolution set to 70,000 FWHM. Analysis was performed in the positive ion mode and L6 and internal standard (I.S.) ions $[M+H]^+$ were monitored at m/z 387.16743 and 373.15168, respectively. The amples were prepared by protein precipitation. 10µL I.S. (1µg/mL) was added to 50µL plasma, followed by 300µL acetonitrile. Samples were vortexed for 30 seconds and centrifuged at 13,000g for 10 minutes. The clear supernatant was then transferred to a glass vial andevaporated under a slight nitrogen stream at 37°C. Finally, the residue was reconstituted in 100µL of mobile phase and 10µL were injected in LC-MS/HRMS.

Evaluation of MS-L6 antitumor efficacy in cell lines derived xenografts (CDX)models:

Experiments were performed by ANTINEO (Lyon, France), a CRO specialized in preclinical oncology. Briefly, 5 x 10^6 RL or SUDHL4 cells per injection (200µL) were first injected

subcutaneously into SCID mice (females, 4 weeks). To circumvent the heterogeneity of tumor growth, 2 x 2mM

pieces of a first tumor were then surgically implanted into the flanks of other animals. This set of mice was used for the efficacy study. Mice were randomized when tumors reached a mean volume of 100mM^3 for the 2 groups (control and L6). All mice were observed to detect any toxic effects of the product. The endpoints are defined by animal ethics as tumor diameter of > 18mm, significant weight loss or changes in animal well-being. To assess the efficacy of the compounds on tumorigenesis, tumor volume was measured three times per week. Primary tumor sizes were measured with calipers and tumor volume (TV) was extrapolated to a sphere using the formula TV= 4/3 π x r3, by calculating mean radius from the two measurements. The median and standard deviation were also calculated for each group. Median is preferred to mean to exclude extreme values. MS-L6 was administered by intraperitoneal injection five times per week, at a dose of 50mg/kg. Control DMSO was also administered by intraperitoneal injection five times per week.

Table of immune populations analyzed

Viable immune populations identified (Annexin negative and CD45 positive)

CD45 is used to assess possible non-leukocyte contamination of the lymphocyte window in the graphical analysis.

**Lymphocytes B** (CD3- / CD19+)			
sub populatio	B transitionnelles	CD10+ CD27-	
	B naives	CD10- CD27-	
	B mémoires	CD10- CD27+	
	B activés	CD80+	
**Lymphocytes T** (CD3+ / CD19-)			
	LyT CD4+	CD4+ CD8-	
		LyTreg : CD25+ CD127low	
		Ly cTFH : CD25low/- et PD1+	
	LyT CD8+	CD4- CD8+	
**Lymphocyte**	naïve	CD45RA+ CCR7+	
sub populatio	EMRA	CD45RA+ CCR7-	
	central mem	CD45RA- CCR7+	
	effecteur mem	CD45RA- CCR7-	
**NK-T**	CD3+ CD56+		
**NK**	CD3- CD19-		
		CD56+ NKp46+	
	early NK	CD56high CD16-	
	mature NK	CD56+ CD16+	
**Monocytes**	morphologie		
	classique	CD14+ CD16-	
	non classique	CD14- CD16+	
	intermediate	CD14+/low CD16+/low	

Cell Marker	Clone	Fluorochrome	Cell Target
Annexin V		FITC	Apoptotic cells
CD3	SK7	APC/Fire810	T cells
CD4	RPA-T4	APC	T cells substets
CD8	SK1	SparkBlue 550	T cells substets
CD10	HI10a	PECF594	B cells subsets
CD11c	3.9	APC-R700	Dendritic cells
CD14	MφP9	PerCP	Monocyte
CD16	3G8	SuperBright 436	Monocyte & NK subsets
CD19	HIB19	APC-H7	B cells
CD25	2A3	BV605	T cells substets
CD27	L128	BV510	B cells subsets
CD45	HI30	PerCPCy5.5	
CD45RA	HI19	PE/Fire700	T cells substets
CD56	NCAM16.2	BV786	NK cells
CD80	L307.4	PE	B cells subsets
CD127	HIL-7R-M21	BV421	T cells substets
CCR7	G043H7	BV711	T cells substets
NKp46	9,00E+02	AF647	NK cells
PD1	EH12.1	PECy7	T cells substets

List of the cell lines used in the study.

Cellosaurus - SIB Swiss Institute of Bioinformatics | Expasy

T lymphoma cell lines	
**Name**	**Cellosaurus ID**
KARPAS384	CVCL_2541
KARPAS299	CVCL_1324
JURKAT	CVCL_0065
SUDHL1	CVCL_0538
DEL	CVCL_1170
OCI-Ly13.2	CVCL_8797
OCI-Ly17	CVCL_8798
MOTN-1	CVCL_2127
SR786	CVCL_1711
SUDHL1	CVCL_0538
L82	CVCL_2098
HUT102	CVCL_3526
DERL2	CVCL_2016
HUT78	CVCL_0337
MYLA	CVCL_M415

RMS cell lines	
**Name**	**Cellosaurus ID**
RH30	CVCL_0041
RD136	CVCL_1649

B lymphoma cell lines	
**Name**	**Cellosaurus ID**
RL	CVCL_1660
KARPAS 422	CVCL_1325
SUDHL6	CVCL_2206
SUDHL4	CVCL_0539
PFEIFFER	CVCL_3326
TMD8	CVCL_A442
SSK41	CVCL_C123
VL51	CVCL_3169
OCI-Ly3	CVCL_8800
NALM6	CVCL_0092
BJAB	CVCL_5711
DAUDI	CVCL_0008

Leukemia cell lines	
**Name**	**Cellosaurus ID**
HL60	CVCL_0002
U937	CVCL_0007
K562	CVCL_0004

List of the chemicals and other products used in the study

Chemicals and others products used in the manuscript

	Manuscript	Name	Supplier	Reference
Media	RPMI	RPMi 1640 (1X) - Glutamax	Thermo Fischer	61870-010
	HPLM	Human Plasma-Like Medium	Gibco	A48991-01
	DMEM	DMEM (1X) - Glutamax	Gibco	31966-021
	Penicilline-Streptomycine	Pen-Strep	Gibco	15140-122
	**DMEM**		PAN Biotech	P04-03500
	**M199**	Medium 199 avec Earle's Salts	Dutscher	L0356-500
	**Glutamine**	L-glutamine 200mM	PAN Biotech	P04-80100
	**BSA**	Albumin fraction V fatty acid free	Roche	10775835001
	Trypsine-EDTA	Trypsine-EDTA (1X)	Gibco	25300-054
	RPMI Seahorse	Seahorse XF RPMI	Agilent	103576-100
	Glucose	Seahorse XF Glucose (1.0 M solution)	Agilent	103577-100
	Glutamine	Seahorse XF Glutamine (200 mM solution)	Agilent	103579-100
	Pyruvate	Seahorse XF Pyruvate (100 mM solution)	Agilent	103578-100
	Cell-Tak Corning	Corning™ Cell-Tak	Fischer Scientific	10317081
Chemicals	MS-L6	MS-L6	CERMN Caen	
	IM156	IM156	MCE (MedChemExpress)	Ref #HY-136093A/CS-0131591
	IACS-010759	IACS-010759		
	Rotenone	Rotenone	Sigma	R8875
	**sucrose**	sucrose	Sigma	S7903
	**Tris-HCl**	Tris hydrochlorid	Roth	9090.3
	**EGTA**	Ethylene glycol-bis(β-aminoethyl ether)-N,N,N′,N′-tetraacetic acid tetrasodiu	Sigma	E4378
	**NADH**	NADH grade II disodium salt	Roche	10128023001
	**KCl**	Potassium chloride	Sigma	P4504
	**digitonin**		Acros organics	40756500
	**malate**	L-malic acid disodium salt	Sigma	M9138
	**glutamate**	L-glutamic acid monosodium salt	sigma	G1626
	**succinate**	sodium succinate dibasic hexahydrate	Sigma	S2378
	**ATP**	Adenosine 5′-triphosphate disodium salt hydrate	Sigma	A2383
	**ADP**	Adenosine 5′-diphosphate sodium salt	Sigma	A2754
	**oligomycin**	Oligomycin from Streptomyces diastatochromogenes	Sigma	O4876
	**inorganic phosphate**	orthophosphoric acid 85%	Fulka	79617
	**TMPD**	N,N,N′,N′-Tetramethyl-p-phenylenediamine dihydrochloride	Sigma	T3134
	**Ascorbate**	(+)-Sodium L-ascorbate	Sigma	A7631
	**DMSO**	Dimethyl sulfoxide	Sigma	D2650
	**KCN**	potassium cyanide	Fulka	60178
	**decylubiquinone**		Sigma	D7911
	**PCA (perchloric acid)**	perchloric acid	Sigma	244252
	**KOH (potassium hydroxide)**	potassium hydroxide	Sigma	P1767
	**MOPS**	4-Morpholinepropanesulfonic acid	Sigma	M1254
	**HCL**	hydrochloric acid	Roth	K025.1
	**Rhodamine 123**		Sigma	R8004
	**FCCP**	Carbonyl cyanide 4-(trifluoromethoxy)phenylhydrazone	Sigma	S2920
	**TMRM**	Tétraméthylrhodamine, ester méthylique, perchlorate	Molecular Probes	T668
	**Mito Tracker Green**	MitoTracker™ Green FM Dye	Molecular Probes	M46750
	**CCCP**	Carbonyl cyanide 3-chlorophenylhydrazone	Sigma	C2759
Kits	Cell Titer Fluor	***CellTiter*** -Fluor™ Cell Viability Assay	Promega	G6082
	Calcein/Biotin	LIVE/DEAD Viability/Cytotoxicity Assay Kit	Invitrogen	L3224
	**Annexin**			
	**PI**			
	Mycoplasma detection	MycoAlert Mycoplasma Detection	LONZA	LT07-318
Animaux	SCID mice	SCID, females, 4 weeks	Charles River	
	Rats	Wistar Han, males	Charles River	RjHan:WI
	NDI1	pLV[Exp]-EGFP:T2A:Puro-EF1A>sce_NDI1	VectorBuilder	Vector ID: VB900123-1673qzj
Plasmids	Control	pLV[Exp]-EGFP:T2A:Puro-EF1A>mCherry		Vector ID: VB010000-9298rtf
